# Alpha-Amino-Beta-Carboxy-Muconate-Semialdehyde Decarboxylase Controls Dietary Niacin Requirements for NAD^+^ Synthesis

**DOI:** 10.1016/j.celrep.2018.09.091

**Published:** 2018-10-30

**Authors:** Laura Palzer, Jessica J. Bader, Frances Angel, Megan Witzel, Sydney Blaser, Alexis McNeil, Miles K. Wandersee, N. Adrian Leu, Christopher J. Lengner, Clara E. Cho, Kevin D. Welch, James B. Kirkland, Ralph G. Meyer, Mirella L. Meyer-Ficca

**Affiliations:** 1 Department of Animal, Dairy, and Veterinary Sciences, College of Agriculture and Applied Sciences, Utah State University, Logan, UT 84332, USA; 2 Utah Experimental Station, Utah State University, Logan, UT 84332, USA; 3 Department of Biomedical Sciences, School of Veterinary Medicine, University of Pennsylvania, Philadelphia, PA 19104, USA; 4 Institute for Regenerative Medicine, University of Pennsylvania, Philadelphia, PA 19104, USA; 5 Department of Nutrition, Dietetics, and Food Sciences, College of Agriculture and Applied Sciences, Utah State University, Logan, UT 84332, USA; 6 Poisonous Plant Research Laboratory, USDA Agricultural Research Service, Logan, UT 84332, USA; 7 University of Guelph, Guelph, ON N1G 2WI, Canada; 8 Lead Contact

## Abstract

NAD^+^ is essential for redox reactions in energy metabolism and necessary for DNA repair and epigenetic modification. Humans require sufficient amounts of dietary niacin (nicotinic acid, nicotinamide, and nicotinamide riboside) for adequate NAD^+^ synthesis. In contrast, mice easily generate sufficient NAD^+^ solely from tryptophan through the kynurenine pathway. We show that transgenic mice with inducible expression of human alpha-amino-beta-carboxy-muconate-semialdehyde decarboxylase (ACMSD) become niacin dependent similar to humans when ACMSD expression is high. On niacin-free diets, these acquired niacin dependency (ANDY) mice developed reversible, mild-to-severe NAD^+^ deffciency, depending on the nutrient composition of the diet. NAD deficiency in mice contributed to behavioral and health changes that are reminiscent of human niacin deficiency. This study shows that ACMSD is a key regulator of mammalian dietary niacin requirements and NAD^+^ metabolism and that the ANDY mouse represents a versatile platform for investigating pathologies linked to low NAD^+^ levels in aging and neurodegenerative diseases.

## In Brief

Palzer et al. show that ACMSD overexpression renders mice dependent on dietary niacin in a manner similar to that of humans. On niacin-free diets, such mice become NAD^+^ deficient. These mice can serve as a model for a range of human diseases such as aging and metabolic diseases that are associated with NAD^+^ deficiency.

## Graphical Abstract

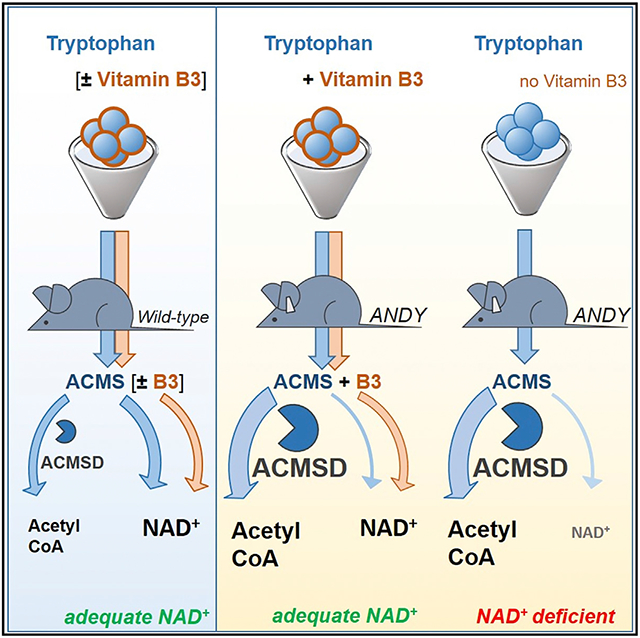

## INTRODUCTION

Humans depend on B3 vitamins (nicotinic acid and nicotinamide), nicotinamide riboside, and tryptophan as dietary precursors for nicotinamide adenine dinucleotide (NAD^+^) synthesis (Kirkland and Meyer-Ficca, 2018). NAD^+^ levels in whole blood can be measured to assess adequate dietary niacin intake (Fu et al., 1989; Jacobson et al., 1999; Tang et al., 2008). NAD^+^ levels vary significantly between individuals, and up to 15%–20% of the general population and 26% of elderly people may be niacin deficient, even in countries with niacin-fortification programs (Jacobson, 1993; Paulionis et al., 2005). Such NAD^+^ deficiency is often not clinically recognized or understood, but potentially highly prevalent and therefore a current research focus. Particularly aging-associated metabolic changes are linked to low NAD^+^ levels (Imai, 2009, 2011; Imai and Guarente, 2014; Rehan et al., 2014). NAD^+^ and the reduced form NADH are coenzymes for redox reactions in metabolic processes such as the Krebs cycle, glycolysis, fatty acid oxidation, gluconeogenesis, and lipid and steroid synthesis. NAD^+^ is also a co-substrate for sirtuins, which are epigenetic regulators, and ADP-ribose transferases. Sirtuins are class III NAD^+^-dependent histone deacetylases that regulate metabolic function, longevity, and aging (Imai and Guarente, 2016; O’Callaghan and Vassilopoulos, 2017). Availability and redox status of NAD^+^ are emerging as major factors in the development of disorders, including metabolic dysfunction linked to aging-related pathophysiology of the circadian rhythm, DNA repair, and cancer (Asher and Sassone-Corsi, 2015; Belden and Dunlap, 2008; Imai, 2010). Variations in NAD^+^ levels may therefore link the metabolic status of a cell to its epigenetic regulatory machinery. The “NAD World” describes this concept (Imai, 2009, 2010, 2016; Imai and Guarente, 2016).

**Figure 1.**
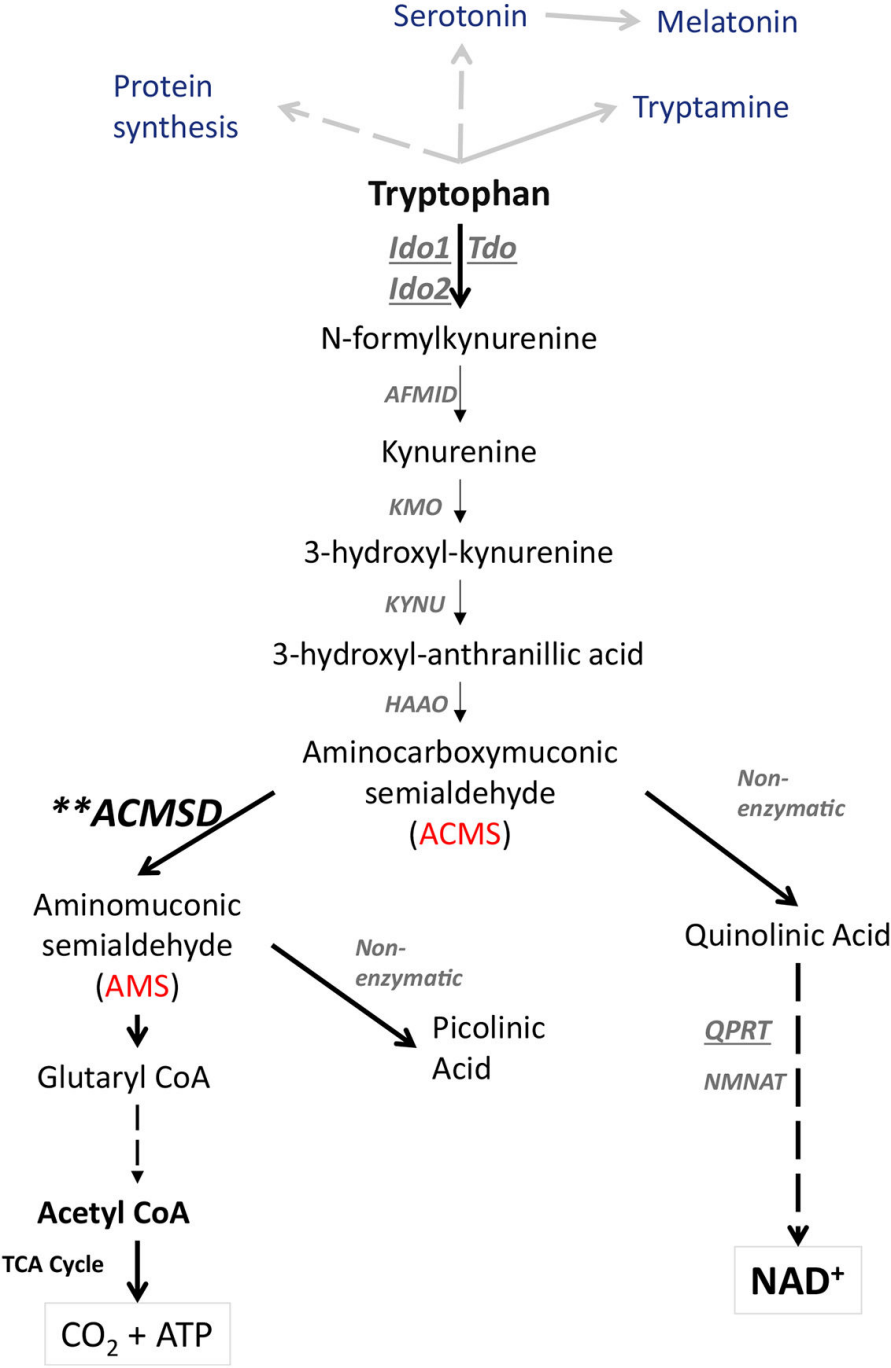
The Principle Underlying Acquired Niacin Dependency by Overexpression of ACMSD In addition to its relevance for serotonin, melatonin, tryptamine, and general protein synthesis, dietary tryptophan can be converted to either NAD^+^ or acetyl-CoA via the kynurenine metabolic pathway in mammals. ACMSD, ACMS decarboxylase [EC:4.1.1.45]; AFMID, *N*-formylkynurenine formamidase [EC:3.5.1.9]; CoA, coenzyme A; HAAO, 3-hydroxyanthranilate 3,4-dioxygenase [EC: 1.13.11.6]; IDO1, indoleamine 2,3-dioxygenase 1 [EC: 1.13.11.52]; IDO2, indoleamine 2,3-dioxygenase 2 [EC:1.13.11.52]; KMO, kynurenine 3-monooxygenase [EC:1.14.13.9]; KYNU, kynureninase [EC:3.7.1.3]; NAD^+^, nicotinamide adenine dinucleotide; NMNAT, nicotinamide mononucleotide adenylyltransferase [EC:2.7.7.1 2.7.7.18]; QPRT, quinolinate phosphoribosyl transferase [EC:2.4.2.19]; TCA cycle, tricarboxylic acid cycle; TDO, tryptophan 2,3-dioxygenase [EC:1.13.11.11].

Modeling NAD^+^ deficiency in rodents has been problematic because they efficiently convert tryptophan to NAD^+^ via the kynurenine pathway (Figure 1) and do not seem to depend as much on dietary niacin as a precursor for NAD^+^ synthesis as humans. Humans need ~60–70 mg of tryptophan to produce the equivalent of the amount of NAD^+^ produced from 1 mg of niacin, if consuming an experimental diet that is tryptophan rich and niacin-free (Fukuwatari et al., 2004; Horwitt et al., 1981). With limited dietary tryptophan, this conversion rate is even lower (Fu et al., 1989). This relatively inefficient tryptophan-to-NAD^+^ conversion rate, when compared to the conversion rate in rodents, makes humans susceptible to developing NAD^+^ deficiency on niacin-free and protein-poor diets. Several knockout mouse models have been generated to disrupt the kynurenine pathway, but these mice either did not become truly NAD^+^ deficient due to enzyme redundancy or the disruptions caused the accumulation of toxic intermediates (Terakata et al., 2012, 2013). Considering alternative strategies, we hypothesized that the differences in rodent and human niacin requirements may be caused by the different efficiency of a key enzymatic step in the *de novo* NAD^+^ synthesis from tryptophan in the kynurenine pathway. In this step, the intermediate tryptophan metabolite alpha-amino-beta-carboxy-muconate-semialdehyde (ACMS) is either decarboxylated by the enzyme ACMS decarboxylase (ACMSD) to alpha-amino-muconate-semi-aldehyde (AMS) or left to undergo spontaneous cyclization to form quinolinic acid. Quinolinic acid then serves as a precursor for NAD^+^ synthesis, whereas AMS spontaneously forms picolinic acid, which is ultimately metabolized to acetyl coenzyme A (acetyl-CoA). The efficiency of NAD^+^ synthesis from tryptophan should therefore hinge on the extent of ACMS accumulation, which is regulated by ACMSD. Low ACMSD activity should permit efficient NAD^+^ synthesis from tryptophan, while high ACMSD activity is expected to prevent efficient tryptophan-to-NAD^+^ conversion. The formation of quinolinate and NAD^+^ is impaired if there is high activity of ACMSD, and this appears to be a main source of variation between species in the efficiency of the conversion of tryptophan to NAD^+^ (Ikeda et al., 1965)

If ACMSD is the gatekeeper that shunts tryptophan to either NAD^+^ or acetyl-CoA synthesis, then high ACMSD activity should result in the dependence of an individual on dietary niacin as a source of NAD^+^, which could be the case in niacin-dependent species such as humans. We generated a transgenic mouse model with inducible human (h)ACMSD overexpression (acquired niacin deficiency, or ANDY, mouse) to test the hypothesis that elevated ACMSD activity renders rodents niacin dependent, which should result in NAD^+^ deficiency on a niacin-free diet. The results show that ANDY mice with ACMSD overexpression depend on dietary niacin intake and that their NAD^+^ levels are tunable by defined diets, ranging from mildly to severely deficient. Our findings suggest that ANDY mice are a powerful and versatile model for a range of investigations into the links between NAD^+^ levels and aging-related diseases, DNA repair, metabolic disease, as well as for dietary research.

## RESULTS

### Generation of Mice with Inducible Overexpression of hACMSD

The ANDY mouse model permits doxycycline (DOX)-inducible expression of human ACMSD to allow for precise control of transgene expression levels (Figure S1A). ANDY mice were generated using mouse embryonic stem cells harboring a single copy of the human *ACMSD* coding sequence under control of the tetracycline operator (TetO) in safe-haven chromatin downstream of the *Col1a1* locus, along with a copy of M2 reverse tetracycline transactivator (M2rtTA) constitutively expressed from the ROSA26 locus. DOX in drinking water elicits robust, dose-dependent gene activation *in vivo* (Beard et al., 2006). The resulting ANDY mice (C57BL/6J-Gt[Rosa]26Sor^tm1[rTTa*M2]Jae^Col1a1^tm6[tetO-ACMSD]MMF^) showed normal fertility and development, were born in normal Mendelian ratios, and exhibited no discernible phenotype in the absence of DOX.

### DOX Administration Induces Robust, Sustained *hACMSD* Overexpression

**Figure 2.**
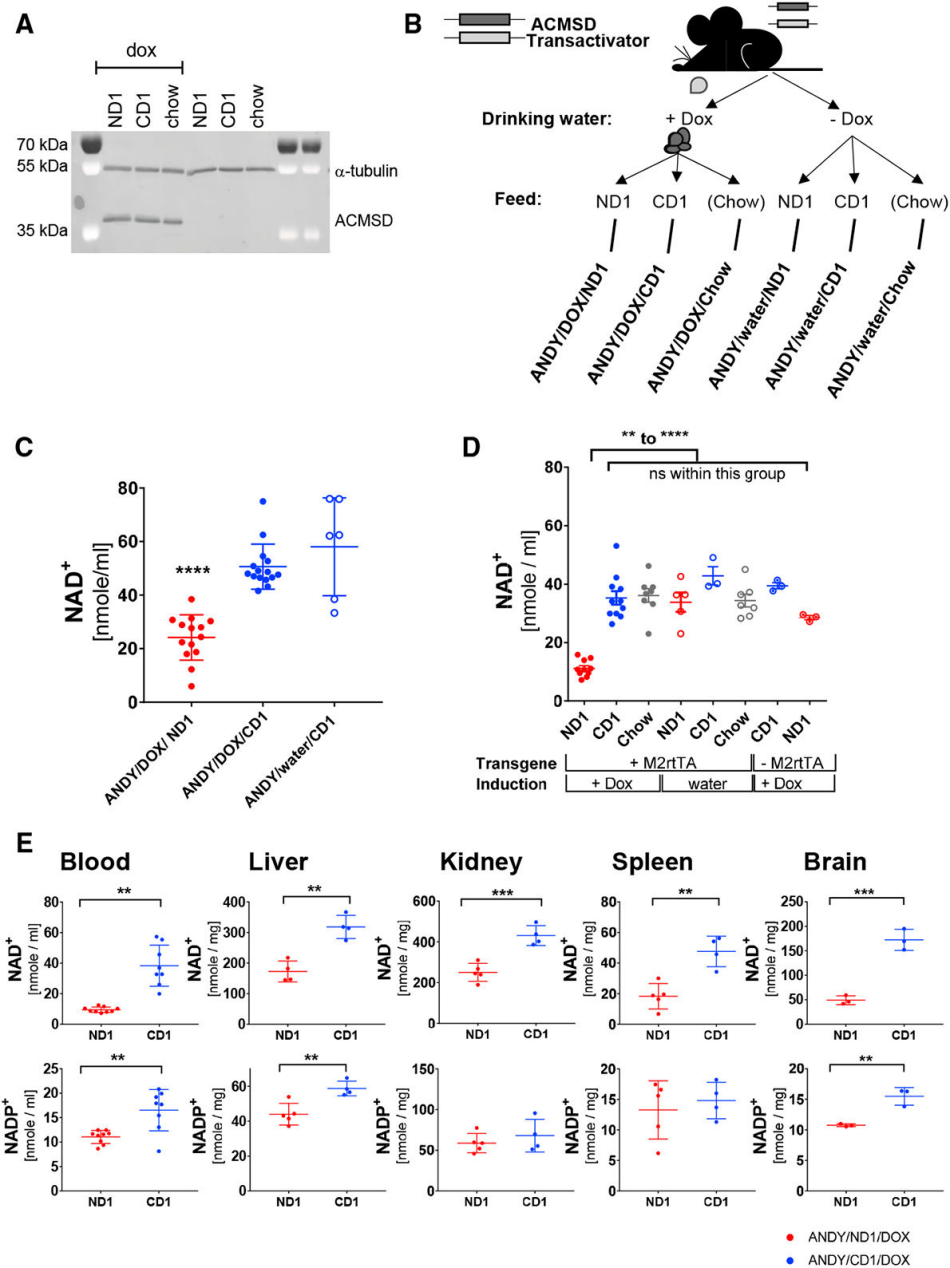
Doxycycline-Inducible Expression of hACMSD Results in Dietary Niacin-Dependent Decrease in NAD^+^ and NADP^+^ Levels (A) ACMSD overexpression remained stable for at least 4 months. Immunoblot analysis of hACMSD expression in liver from male transgenic ANDY littermates after 4 months on defined diets and continuous DOX administration. Different niacin content in feed did not affect transgene expression. Alpha-tubulin served as loading control. (B) Design of feeding trials. DOX administration induced hACMSD transgene expression (Dox); animals without ACMSD expression were controls (water). Within each group, equal numbers of mice were assigned to a niacin-depleted diet (ND1), a niacin-replete but otherwise identical diet (CD1), or chow feed (chow; diet compositions in Tables S1 and S2). (C and D) Reduced blood NAD^+^ in male ANDY mice after 3 weeks (C) and 12 weeks (D) on niacin-free diet (ND1) compared to controls without hACMSD overexpression or on niacin-replete feed (CD1, chow). Data on ANDY/DOX/ND1 and ANDY/DOX/CD1 mice are from 4 and 3 independent experiments in (C) and (D), respectively. All dataata represent mean ± SEM. (E) Lower NAD^+^ levels in ANDY mice after 10 weeks on ND1 in all tissues tested. NADP^+^ levels were also lower, with significant differences only seen in blood, liver, and brain. Dots represent individual mice in (C)–(E): red dots are ANDY/Dox/ND1, blue dots are ANDY/DOX/CD1. One-way ANOVA, Tukey’s multiple comparison test. All data represent mean ± SD. **p < 0.01, ***p < 0.001. See also Figures S1, S2, and S3.

Administration of DOX induced hACMSD expression from the transgene within 48 hr (Figure S1B). As long as DOX was administered, expression was maintained, with 4 months being the longest time interval tested (Figure 2A). In addition, transgene expression levels depended on hemi- or homozygosity of the *hACMSD* and *M2rtTA* transgenes. ACMSD amounts were highest in ANDY mice homozygous for both transgenes, while hemizygosity of either the *hACMSD* or *M2rtTA* alleles (or both) resulted in reduced levels (Figure S2A). All subsequent investigations used ANDY mice homozygous at both the *hACMSD* and *M2rtTA* loci.

### Acquired Niacin Dependency in Mice with hACMSD Overexpression

To evaluate dietary niacin requirements for maintaining blood NAD^+^ levels, male ANDY mice were kept on either a niacin-free diet (ND1) or the same diet supplemented with 30 mg/kg niacin (CD1), or regular chow. Combining these diets with DOX-induced transgene expression or water-only controls resulted in six different treatment groups (ANDY/DOX/ND1, ANDY/DOX/CD1, ANDY/DOX/chow, ANDY/water/ND1, ANDY/water/CD1, ANDY/water/chow; Figure 2B). Animals lacking the *M2rtTA* transactivator necessary for ACMSD expression on ND1 and CD1 diets provided additional controls. After 3 weeks on the experimental diets, blood NAD^+^ contents were significantly decreased in hACMSD-expressing ANDY mice on a ND1 (ANDY/DOX/ND1) relative to ANDY animals on the niacin-replete diet CD1 (ANDY/DOX/CD1) (Figure 2C). Additional controls at the 4-month point showed that the decreased NAD^+^ status depended on the combination of ND1 and *hACMSD* overexpression. NAD^+^ levels in animals on a niacin-replete control diet (CD1) or regular chow or in animals that did not overexpress *hACMSD* due to the absence of DOX or the M2rtTA were not significantly affected (Figure 2D). Of note, NAD^+^ values were significantly reduced in the blood and tissues of niacin-deficient ND1-fed animals that overexpress hACMSD (+DOX) compared to ND1-fed ANDY mice not overexpressing *hACMSD* (water, −DOX) (Figures 2D and S2B). NAD^+^ phosphate (NADP^+^) remained stable until 3 weeks on a niacin-deficient diet (Figure S2C), but mice on an ND1 diet ultimately became NADP^+^ deficient as well (Figure S2D). In line with the reduced blood NAD^+^ contents, NAD^+^ was also reduced in liver, kidney, spleen, and brain of ANDY mice on ND1 diet (Figure 2E). NADP^+^ values were also lower in these tissues, but differences were only significant in liver, blood, and brain, and not kidney and spleen. Blood NAD^+^ values decreased similarly in both male and female ANDY mice on a niacin-deficient diet (Figures S3A and S3B).

The data indicate that in the absence of hACMSD overexpression, mice do not become NAD^+^ deficient on an ND1, suggesting that rodents efficiently metabolize tryptophan to NAD^+^ via the *de novo* pathway. In contrast, elevated expression of *hACMSD* in the ANDY mice resulted in a dependency on dietary niacin for the synthesis of NAD^+^. *hACMSD* overexpression alone did not cause NAD^+^ deficiency in animals on the niacin-replete CD1 diet or regular chow. Results from ANDY mice and from controls lacking the *M2rtTA* allele provide evidence that neither M2rtTA expression nor DOX administration alone affect NAD^+^ levels in the absence of the *hACMSD* transgene. These data support the hypothesis that elevated expression of *hACMSD* results in the dependency of animals on dietary niacin to maintain normal NAD^+^ and NADP^+^ levels.

### NAD^+^/NADH and NADP^+^/NADPH Deficiency in ANDY Mice Is Reversible

**Figure 3.**
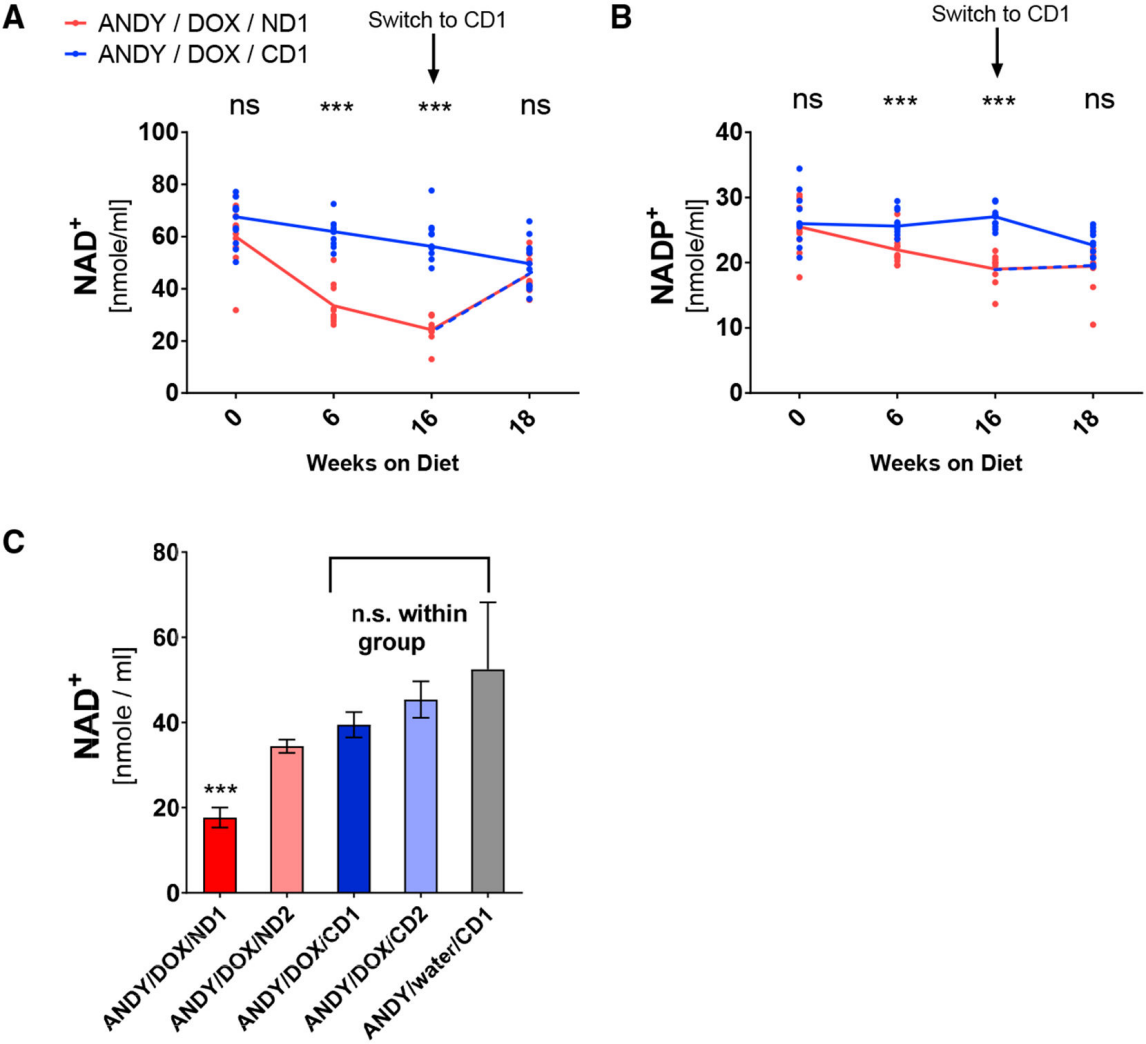
NAD^+^ Deficiency in Animals with hACMSD Expression Depends on Niacin and Protein Content in the Diet and Is Reversible (A and B) Blood NAD^+^ (A) and NADP^+^ (B) levels declined over time in male ANDY/DOX/ND1 mice, but not in ANDY/DOX/CD1 mice. NAD^+^ and NADP^+^ adjusted to control levels within 2 weeks after switching NAD^+^-deficient ANDY/DOX/ND1 animals to niacin-replete CD1; n = 11 mice per group. Significance was tested by multiple t tests with Benjamini two-stage linear step-up (Tables S3 and S4). (C) Blood NAD^+^ levels of male ANDY/DOX mice depended on the niacin and protein content in the diets that the animals received for 12 weeks (Tables S1 and S2). Blood NAD^+^ content was lowest in ANDY/DOX/ND1 on the niacin-free, low-protein diet (ND1). ANDY/DOX/ND2 (on niacin-free, but higher protein ND2) had slightly higher blood NAD^+^ content. Blood NAD^+^ values of ANDY/DOX/CD1 and ANDY/DOX/CD2 (on diets with low protein and 30 mg/kg niacin [CD1], or high protein and 60 mg/kg niacin [CD2]) were not significantly different from values measured in ANDY/water/CD1 (without hACMSD transgene expression). Data represent mean ± SEM; one-way ANOVA and Tukey’s multiple comparison test; n = 6–10 per group. See also Figure S4.

To determine the kinetics of declining NAD^+^ and NADP^+^ levels and the reversibility of the observed NAD^+^ deficiency, we monitored blood NAD^+^/NADH and NADP^+^/NADPH levels over time in ANDY/DOX/ND1 and ANDY/DOX/CD1 mice. Total blood NAD^+^ and NADP^+^ were both significantly lower after 6 weeks and after 16 weeks on ND1 (Figures 3A and 3B). ND1-fed animals were then put on the niacin-replete CD1 diet. Two weeks after switching NAD^+^-deficient mice to the niacin-replete CD1 diet, blood NAD^+^ and NADP^+^ values had recovered and became indistinguishable from animals continuously fed the CD1 diet for 18 weeks. Animals on the CD1 diet also had a mild but reproducible and significant decline in blood total NAD^+^ and NADH during the 16-week course of the feeding trials, which may be due to a natural age-related decline in NAD^+^, similar to what has been described in humans (Braidy et al., 2011; Zhu et al., 2015) (Figures S4A and S4B), to adaptation to the diets, or to the development of the mice.

Declines in both nucleotide pools in ANDY/DOX/ND1 mice call into question whether the ratio between NAD^+^ and NADP^+^ or the total NAD^+^/NADH pool alone is the most sensitive indicator of niacin deficiency. In clinical use, the ratio has the important advantage of being independent of sample size. The decline in blood total NADP^+^ was less pronounced than the concomitant lowering of total NAD^+^/NADH, but statistically significant. These data support the notion that blood NADP^+^ levels are more tightly controlled and fluctuate less than NAD^+^ levels (Fu et al., 1989), and they may indicate that NAD^+^ depletion in the ANDY/DOX/ND1 groups was severe. In ANDY/DOX/ND1 mice, overall niacin indices were reduced to <50% of controls, which would be considered severe in humans (Figures S2D and S2E).

To determine the extent to which tryptophan-to-niacin conversion is decreased in the ANDY model, a second diet (ND2) was formulated with 20% casein (twice the tryptophan of ND1). A niacin-replete diet with matched casein content served as a second control (CD2) (Table S2). Blood NAD^+^ levels in ANDY/DOX animals on ND2 (ANDY/DOX/ND2) for 12 weeks were lower than NAD^+^ levels in animals on either niacin-replete control diet (ANDY/DOX/CD2 and ANDY/DOX/CD1), but less reduced than in ANDY/DOX/ND1 (Figure 3C), which shows that ANDY/DOX mice are still able to convert a small proportion of tryptophan to NAD^+^.

In summary, the data indicate that the reduced blood NAD^+^/NADH and NADP^+^/NADPH levels in *hACMSD*-overexpressing mice lacking dietary niacin return to normal upon dietary niacin intake. The data also provide evidence that limiting *de novo* NAD^+^ synthesis from dietary tryptophan via the kynurenine pathway further exacerbates the NAD^+^ and NADP^+^ deficiencies caused by dietary niacin restriction. Thus, diets with defined niacin and tryptophan content can be used to reduce blood NAD^+^ in ANDY mice to defined levels, ranging from mild deficiency on the ND2 diet to severe deficiency on the ND1 diet.

### hACMSD Overexpression Results in Elevated Acetyl-CoA Levels

**Figure 4.**
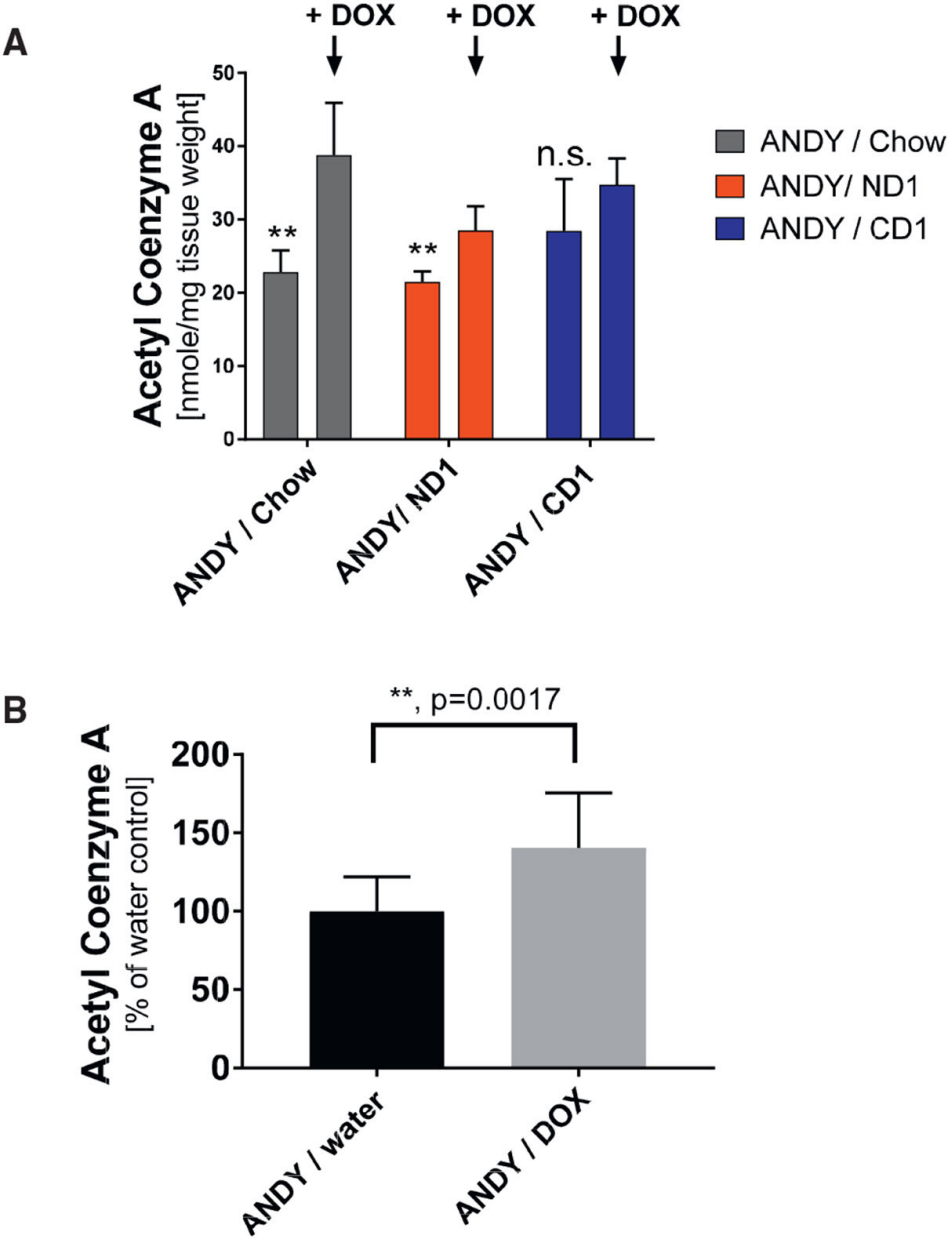
hACMSD Overexpression Resulted in Elevated Acetyl-CoA Contents in Liver (A) Increased liver acetyl-CoA in male ANDY/DOX after 10 days of doxycline administration (arrow) compared to ANDY/water animals. Acetyl-CoA increases upon hACMSD overexpression on chow, ND1, and CD1. Student’s t test; n = 4–5 per group. (B) Acetyl-CoA increases were independent of feed and associated NAD^+^ status. Data in (A) were thus summarized to compare hACMSD transgene induction (ANDY/DOX) to controls (ANDY/water), showing that hACMSD overexpression caused a 1.5-fold acetyl-CoA increase. Student’s t test; n = 13 per group. **p < 0.01; ns, not significant. All data represent mean ± SD. See also Figure S3.

Hepatic acetyl-CoA levels were significantly increased in all animals with induced hACMSD expression, regardless of diet (ND1, CD1, or chow diet) (Figure 4A), suggesting that a significant portion of acetyl-CoA may have been generated by tryptophan metabolism, but much of the acetyl-CoA could also stem from sources other than tryptophan. ANDY/DOX mice receiving standard chow had the highest acetyl-CoA levels, which may be explained by the higher casein, and therefore tryptophan, content in chow (20% instead of 10% in the CD1 and ND1 diets; Table S2). On average, hACMSD overexpression caused a 50% increase in liver acetyl-CoA (Figure 4B).

### Altered NAD^+^/NADH Ratio and Redox State in NAD^+^-Deficient ANDY Mice

**Figure 5.**
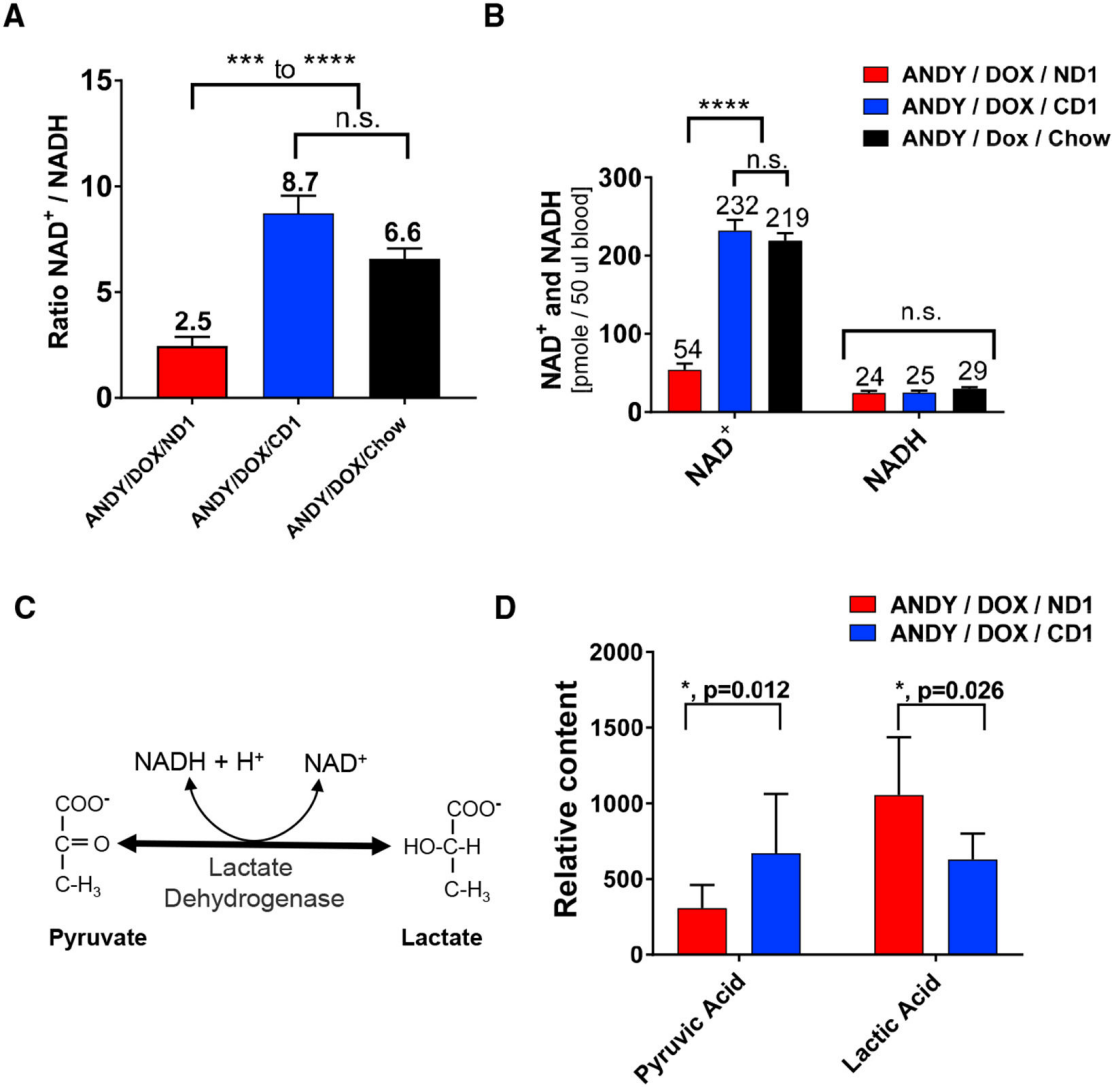
Niacin Deficiency in ANDY Mice Shifted Blood NAD^+^ to NADH/H^+^ Ratio (A) Ratio of oxidized NAD^+^ to reduced NADH in males on indicated feed group for 8–12 weeks. (B) Oxidized NAD^+^ content was lower in male ANDY/DOX/ND1 animals, while NADH values were unchanged. One-way ANOVA and Tukey’s multiple comparison test, n = 7 mice per group. (C) The equilibrium of pyruvate and lactate concentrations depends on the NAD^+^ to NADH ratio. (D) Comparative GC-MS analysis of liver tissues showed lower pyruvic acid content and increased lactic acid content in ANDY/DOX/ND1 compared to ANDY/DOX/CD1. Student’s t test with Welch’s correction for unequal variance; n = 7–9. *p < 0.05, ***p < 0.001, ****p < 0.0001; ns, not significant. All data represent mean ± SEM.

As a coenzyme, NAD^+^/NADH is involved in redox reactions in which NAD^+^ is the oxidizing form and NADH is the reducing form. The total NAD^+^/NADH ratio is estimated to normally range from 3 to 10 in mammals (Lin and Guarente, 2003), which is consistent with results obtained from ANDY/DOX/CD1 and ANDY/DOX/chow controls (Figure 5A). In hACMSD-overexpressing ANDY/DOX/ND1, the NAD^+^/NADH ratio was significantly reduced. The low ratio was due to low NAD^+^ but not NADH levels, which remained normal (Figure 5A).

### NAD^+^ Deficiency Is Associated with Lower Pyruvate and Elevated Lactate Levels

A metabolomics approach to characterize standard liver metabolites in ANDY and control mice indicated a significantly altered pyruvate-to-lactate ratio in the ANDY mice. In the presence of normal levels of the oxidizing coenzyme NAD^+^, lactate dehydrogenase activity converts lactate to pyruvate (Figure 5C). We quantified the ratio of the two metabolites using comparative gas chromatography-mass spectrometry (GC-MS) analyses of livers from niacin-deficient ANDY/DOX/ND1 and niacin-replete ANDY/DOX/CD1 mice. Livers from niacin-deficient animals contained significantly less pyruvate than those from CD1-fed animals (Figure 5D). In addition, niacin-deficient mice had increased lactate levels compared to CD1 control animals. Taken together, the data indicate that NAD^+^ deficiency and the associated shift in the hepatic NAD^+^/NADH redox status in ANDY/DOX/ND1 mice led to a change in the pyruvate-to-lactate balance.

### NAD^+^-Deficient Mice Lose Weight and Become Lethargic

**Figure 6.**
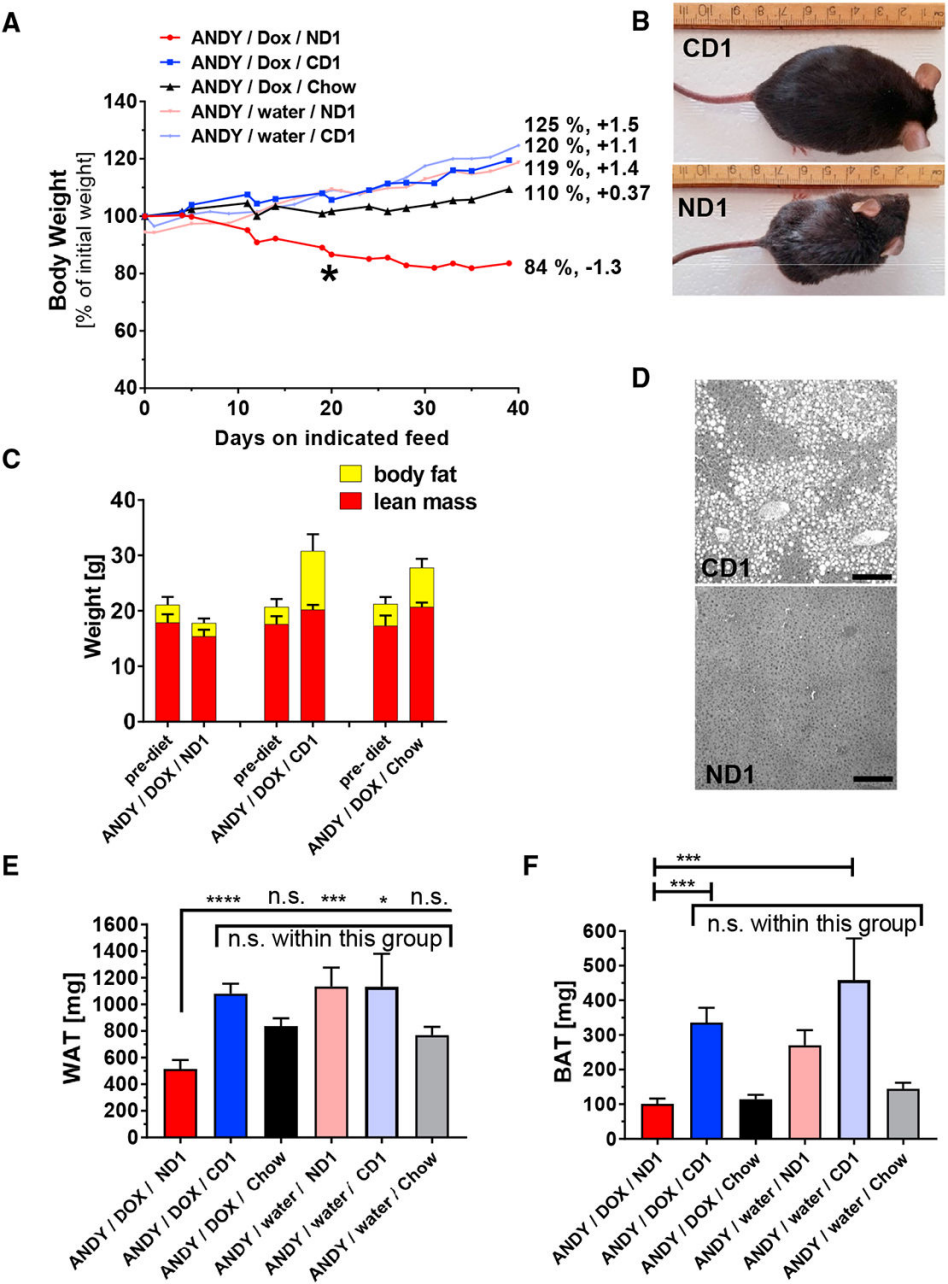
Altered Body Weight and Composition in Male Niacin-Deficient Mice (A) ANDY/DOX/ND1 mice (on niacin-free ND1) had a significant trend to lose body weight (negative slope of −1.1 in post-test for linear trend, p < 0.0001). ANDY/DOX/CD1 (on niacin-replete CD1) and ANDY/DOX/chow (chow) both had a significant trend for weight gain (slope +1.1, p < 0.0001, and slope 0.37, respectively). Without transgene induction (ANDY/water/ND1 and ANDY/water/CD1), both niacin-deficient and niacin-replete diets caused significant weight gain (+1.4 and +1.5). Numbers are body weight after 40 days on feed, as a percentage of starting body weight. Repeated-measurements 1-way ANOVA, post-test for linear trend; a representative experiment of four is shown; n = 4 per group. *First significant difference between ANDY/DOX/ ND1 and other feed groups in individual t tests. (B) Representative ANDY/DOX/CD1 and ANDY/DOX/ND1 animals on CD1 or ND1 for 12 weeks. (C) Body mass composition, shown as fat and lean mass, at start (left columns) and end (right) of a 12-week feeding trial. Body fat of ANDY/DOX/CD1 and ANDY/DOX/chow increased significantly (p < 0.0001 and p = 0.0037, respectively). ANDY/DOX/ND1 did not gain any body fat. All feed groups had small but significant changes in lean body mass. CD1- and chow-fed mice gained lean mass; niacin-deficient ND1 mice lost lean mass. Body composition measured by EchoMRI analyzer; n = 4–7 per group. Data represent mean ± SD. (D) H&E staining of liver from ANDY/DOX/CD1 and ANDY/DOX/ND1 shows hepatic lipid accumulation (fatty liver) in CD1- but not ND1-fed mice. Grayscale image; scale bar, 200 μm. (E) Significantly less epididymal fat in ANDY/DOX/ND1 compared to controls. (F) Significantly less interscapular brown fat in ANDY/DOX/ND1 compared to controls. One-way ANOVA analyses, Tukey’s multiple comparison tests. (E and F) All data represent mean ± SEM. *p < 0.05, ***p < 0.001, ****p < 0.0001; ns, not significant.

ANDY/DOX/ND1 mice invariably lost weight during the course of the 14-week feeding trials (Figure 6A). This effect was more pronounced in males than in females (Figure S3C), which was surprising because females experienced a similar degree of total NAD^+^ deficiency (Figures S3A and S3B). In contrast to the niacin-deficient group, mice fed CD1 or regular chow gained between 10% and 25% of their starting body weight during the same time interval. CD1-fed mice (ANDY/DOX/CD1 and ANDY/water/CD1) and ANDY/water/ND1 showed a tendency to become obese, likely due to the proportionally higher carbohydrate content of CD1 and ND1 diets compared to normal chow (Figures 6A and 6B). As early as 3 weeks into the feeding trial, body weights differed significantly between ANDY/DOX/ND1 and ANDY/DOX/CD1 mice (Figure 6A, *).

Mice on chow had growth curves typical of C57BL/6J mice (see C57BL/6J growth chart at https://www.jax.org/jax-mice-and-services/strain-data-sheet-pages/body-weight-chart-000664) and body weights consistent with animal age. Niacin-deficient ANDY/DOX/ND1 was the only group with a downward trajectory of body weight development. Liver tissue from ANDY/DOX/CD1 mice exhibited histological changes consistent with obesity (fatty liver), while the livers of NAD^+^-deficient mice ANDY/DOX/ND1 were devoid of fat deposits (Figure 6D). MRI analyses of the body composition of ANDY/DOX mice at the end of the feeding trial on ND1, CD1, and chow diets showed a loss of body fat and a minor variation in lean body mass in NAD^+^-deficient animals, while mice on CD1 and chow gained body fat and lean mass (Figure 6C). Of note, all groups started the feeding trial with animals of comparable body composition and age distribution. At the termination of each trial, niacin-deficient ANDY/DOX/ND1 mice had accumulated less white adipose tissue (WAT; average epididymal fat pad weight) than ANDY/DOX/CD1 or ANDY/DOX/chow mice (Figure 6E). WAT weight did not differ significantly between CD1 and chow control groups despite the fact that CD1 mice tended to become obese. ANDY/water/ND1 mice without hACMSD transgene expression had WAT similar to CD1 mice. We concluded that hACMSD transgene expression and the resulting NAD^+^ deficiency in ANDY/DOX/ND1 mice may cause an inability to accumulate body fat. In addition, ANDY/DOX/ND1 mice had the smallest amounts of brown interscapular brown adipose tissue (BAT), together with mice on chow (Figure 6F). ANDY/DOX/CD1 mice had approximately three times larger BAT pads than ANDY/DOX/mice on ND1 or chow. BAT amounts in ANDY/water/ND1 (not overexpressing hACMSD) were comparable to CD1 mice and were 2.5-fold higher than ANDY/DOX/ND1 with transgene expression, again indicating that a lack of niacin in the diet alone did not significantly affect fat metabolism unless the mice simultaneously overexpressed hACMSD. Changes in calorie intake could be ruled out as a cause for the body weight changes between feeding groups over time. Daily calorie intake averaged ~12 kcal/day (Figure S3D). Compared to chow (32%), ND1 and CD1 are relatively protein-poor (10%) and carbohydrate-rich diets (73% versus 54% in chow) with a similar fat content (16.8% versus 14% in chow). Food intake only decreased as mice became severely niacin deficient (Figure S3E), presumably secondary to behavioral changes. Varying degrees of anorexia are common for micronutrient deficiencies in rodents, which may be a protective mechanism against a more rapid metabolic decline with increased feeding on deficient diets. Feed efficiency in ND1- and CD1-fed animals was initially similar but dropped to negative values in ANDY/DOX/ND1, when animals had become NAD deficient after several weeks on the ND1 diet (Figure S3F).

**Figure 7.**
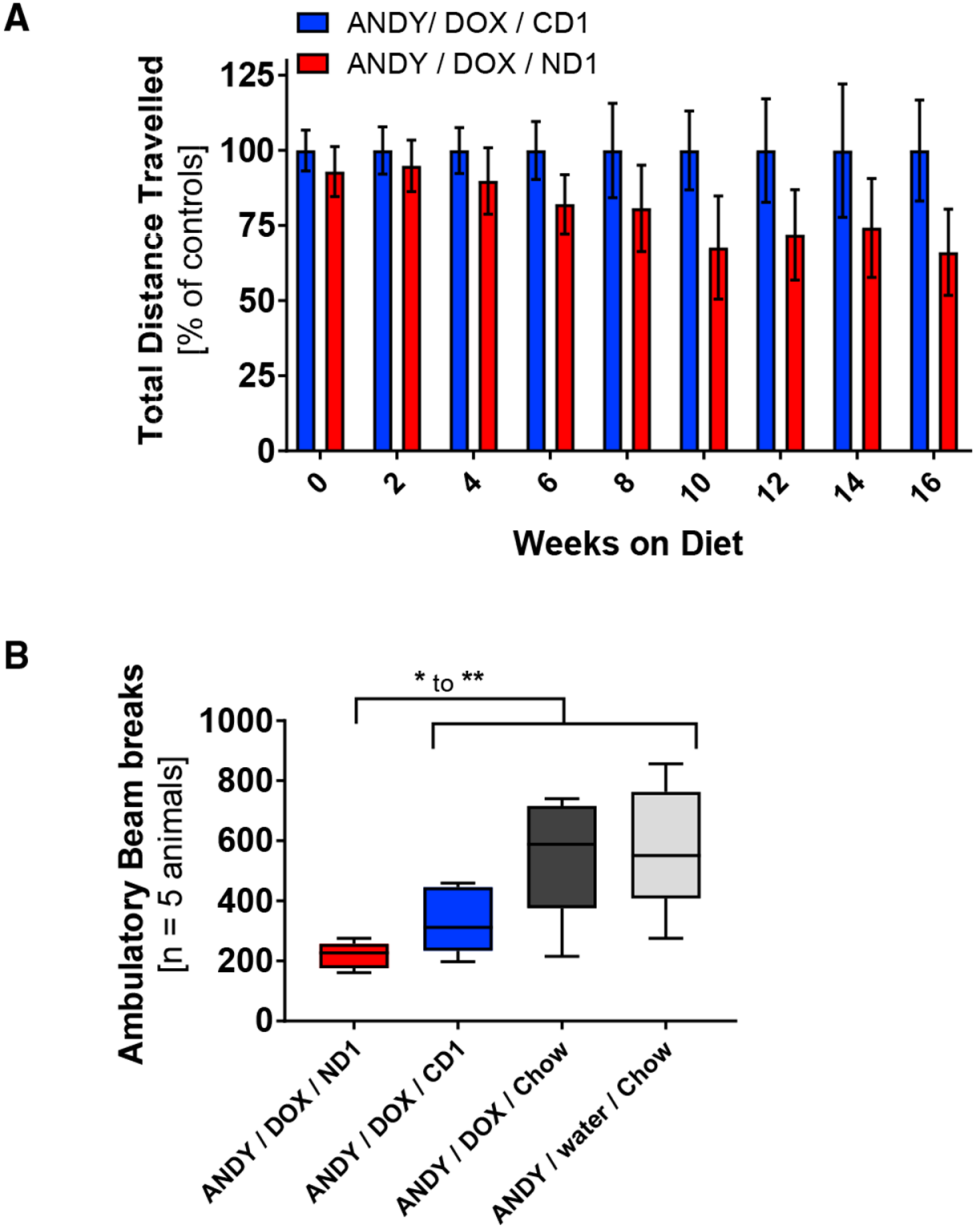
Activity Levels Drop over Time with Progressing Niacin Deficiency The physical activity of animals was measured as distance traveled in an open field test (m/5 min) and as number of ambulatory beam breaks. (A) ANDY/DOX/ND1 distance traveled dropped significantly over time on diet compared to ANDY/DOX/CD1. One-way ANOVA with post-test for linear trend indicated a significant linear decline of activity (slope of −3.658, p = 0.034) in NAD^+^-deficient males compared to controls. Data represent mean ± SEM. (B) Less locomotion indicated by fewer ambulatory beam breaks in NAD^+^-deficient males compared to controls; n = 5 per group; Student’s t test and 1-way ANOVA. Data represent box and whisker blots with Tukey’s whiskers. *p < 0.05, **p < 0.01.

In line with the neurological symptoms (lethargy, apathy, and depression) seen in NAD^+^-deficient humans (Kirkland and Meyer-Ficca, 2018), NAD^+^-deficient mice became lethargic and lacked activity. Compared to ANDY/DOX/CD1 mice, NAD^+^-deficient ANDY/DOX/ND1 mice trended toward lower activity in an open field test, which measures the distance a mouse travels to avoid being out in the open (Figure 7A). Compared to control animals, NAD^+^ deficient mice showed reduced voluntary ambulatory activity (Figure 7B).

## DISCUSSION

The results of this study support the hypotheses that increased ACMSD activity within the kynurenine pathway causes dependency on dietary niacin intake in a murine gain-of-function model and that ACMSD activity is a source of variation between species and individuals in the efficiency of conversion of tryptophan to niacin (Ikeda et al., 1965).

The finding that ANDY mice can be rendered NAD^+^ deficient to model human NAD^+^ deficiency is of interest because NAD^+^ is both an essential coenzyme for energy metabolism and a co-substrate for NAD^+^-consuming enzymes mediating posttranslational protein modifications, such as sirtuins and ADP-ribose transferases (Imai and Guarente, 2014). Due to these varied functions, cellular NAD^+^ may link nutritional status and metabolic processes with the activity of epigenetic modulators. In addition, NAD^+^-dependent enzymes are involved in various human diseases (e.g., cancer, neurodegeneration, multiple sclerosis, Alzheimer disease, Huntington disease) and in aging and longevity. This makes NAD^+^ metabolism an attractive target for drug discovery (Yoshino et al., 2018).

The experimental evidence obtained from ANDY mice highlights ACMSD as a master regulator at the intersection of acetyl-CoA and NAD^+^ metabolism from tryptophan in the kynurenine pathway. Expression of the hACMSD transgene in ANDY mice increased hepatic acetyl-CoA levels by 50%. This suggests that although several different metabolic pathways form acetyl-CoA, the conversion from tryptophan may provide a significant portion of hepatic acetyl-CoA when hACMSD activity is high. Alternatively, low NAD^+^ and NADP^+^ availability may reduce the flux of metabolic pathways that consume acetyl-CoA, which could indirectly cause acetyl-CoA accumulation. The exact mechanisms causing the observed acetyl-CoA increase remain to be determined. These results are consistent with the hypothesis that ACMSD is a key enzyme that regulates the alternative processing of tryptophan to either NAD^+^ or acetyl-CoA, and overexpression of ACMSD results in reduced NAD^+^ levels when niacin intake is low.

Elevated hACMSD expression alone did not change NAD^+^ levels, except in animals fed the niacin-free ND1 diet, but not in CD1- or chow-fed controls (Figure 2). In addition, there was no discernible phenotype of hACMSD expression itself in animals that were NAD^+^ replete. Due to the absence of well-standardized methods for picolinate and quinolinate quantification, those intermediates were not measured. If elevated hACMSD expression altered picolinate or quinolinate production, then it did not result in an outward phenotype in the control animals. Picolinate, unlike quinolinate, has not been clearly identified as neurotoxic, but instead may have a physiological role in maintaining the balance between neurotoxic and neuroprotective kynurenine pathway metabolites (Grant et al., 2009). No toxic effects of hACMSD overexpression or of DOX intake were seen in control animals not fed a niacin-deficient diet, even with higher tryptophan uptake in animals on chow with its higher protein content.

The endogenous modulation of ACMSD activity can change hepatic NAD^+^ levels (Shin et al., 1999). Glucocorticoids and high-protein diets naturally upregulate ACMSD enzyme activity in wild-type rodents, and polyunsaturated fatty acids and a low-protein diet downregulate it (Egashira et al., 2004; Fukuoka et al., 2002). ACMSD activity appears to be controlled at the transcriptional level. Peroxisome proliferator-activated receptor alpha (PPARα) negatively regulates transcription of nuclear factor 4 alpha, which normally maintains ACMSD expression (Shin et al., 2006). Peroxisome proliferators thus increase hepatic NAD^+^ by inhibiting ACMSD expression. In the ANDY mouse, DOX induces hACMSD overexpression independent of endogenous regulators. Increased hACMSD levels result in the opposite effect, which is decreased NAD^+^ formation from tryptophan. Findings of the present study are therefore consistent with studies of endogenous ACMSD activity regulation. It has been shown that tryptophan and total nitrogen intake in mice increase ACMSD activity to form mostly acetyl-CoA. Mice eating 40%–70% protein had extremely high ACMSD activity, and tryp-tophan-to-nicotinamide conversion was extremely low (Shibata, 2018). The ANDY mouse may mimic a situation of a high-protein diet, perhaps exacerbated by high carbohydrate intake, but in the absence of niacin intake. The resulting tryptophan-to-nico-tinamide conversion is therefore low.

The ANDY mouse should enable future investigations into the pathophysiology of NAD^+^ deficiency because it is a tractable small rodent model. Rats can temporarily become niacin deficient when kept on a gelatin/casein-based diet low in total protein (strongly limiting tryptophan intake) and free of niacin from weaning age on. The importance of niacin for genomic stability and for efficient DNA repair upon exposure to alkylating cytotoxic agents was demonstrated in such a model (Boyonoski et al., 1999; Kirkland, 2012; Rawling et al., 1994; Spronck and Kirkland, 2002). The NAD^+^ depletion observed in niacin-deficient rats led to genomic instability in part because NAD^+^ is the substrate for poly(ADP-ribose) polymerases PARP1 and PARP2, which are involved in DNA strand break repair and epigenetic chromatin regulation. Dietary niacin deficiency in young rats also reduced levels of cyclic ADP-ribose formed from NAD^+^ by CD38 and consequently altered learning (Young et al., 2007). The transient NAD^+^ deficiency achievable by such a gelatin-based diet in rats is limited to a short pre-pubertal period, which limits the versatility of such models and makes long-term studies (e.g., aging-related studies) difficult or impossible (Boyonoski et al., 1999; Rawling et al., 1994; Young et al., 2007). Furthermore, rats are less amenable to genetic modification than mice, and therefore there is a paucity of genetic tools available in rats. This further highlights the versatility of the ANDY mouse, which can be crossed to the plethora of widely available reporter and conditional alleles for the study of NAD^+^ biology and metabolism.

Another advantage of increasing ACMSD activity to generate niacin dependency over a knockout approach, in which enzymes involved in the kynurenine pathway are deleted, is that it avoids the accumulation of toxic intermediates such as quinolinic acid. ACMSD activity depletes ACMS, the intermediate in the kynurenine pathway that is directed to NAD^+^ synthesis and thereby prevents ACMS accumulation (Fukuoka et al., 2002; Fukuwatari et al., 2002, 2004). Very low ACMSD activity can cause the accumulation of ACMS and subsequently of quinolinic acid. Quinolinic acid is an excitotoxic metabolite that can cause or contribute to neurodegenerative processes such as epilepsy, Huntington disease (Beal et al., 1986; Feldblum et al., 1988), Parkinson disease (Martí-Massó et al., 2013), and vulnerability to suicidal behavior in humans (Brundin et al., 2016). The need to prevent the accumulation of neurotoxic intermediates such as quinolinic acid likely caused evolutionary pressures favoring high ACMSD activity in humans, which in turn increases pellagra susceptibility. Quinolinic acid is metabolized by quinolinic acid ribosyltransferase (QPRT) to form nicotinic acid mononucleotide (NAMN). Transfer of an adenylate moiety to NAMN forms nicotinic acid adenine dinucleotide (NaAD). Amidation of the nicotinic acid moiety in NaAD to nicotinamide (Nam) finally forms NAD^+^. Besides ACMSD, QPRT therefore represents another rate-limiting step in the tryptophan-to-NAD^+^ *de novo* synthesis pathway. Ablation of the *Qprt* gene in a mouse model resulted in quinolinic acid levels that were elevated >20-fold (Shibata, 2015; Terakata et al., 2012). Several other genetic mouse models aimed at disrupting NAD^+^ synthesis from the kynurenine pathway (Figure 1) have been generated. Genetic deletion of the hepatic enzyme tryptophan 2,3-dioxygenase (TDO; Figure 1), which forms N-formylkynurenine from tryptophan as the first and rate-limiting step of the entire kynurenine pathway, still allowed mice to maintain adequate NAD^+^ levels, likely through the activity of indoleamine 2,3-dioxygenase (IDO) activity (Terakata et al., 2013). The activity of IDO may have been able to compensate for the loss of TDO in the mutant mice, at least with respect to the maintenance of NAD^+^ synthesis for energy metabolism. A genetic deletion of IDO affects the immune response in mice and a number of physiological responses (Baban et al., 2004). A second IDO was discovered, IDO2 (Ball et al., 2007; Metz et al., 2007), and TDO, IDO1, and IDO2 have common and distinct complex functions (Ball et al., 2014). The important functions of these three enzymes, besides the initial metabolism of tryptophan in the kynurenine pathway, create complex phenotypes in mice with disruptions of these genes (Ball et al., 2014; Larkin et al., 2016; Too et al., 2016). Similarly, the disruption of nicotinamide phosphoribosyltransferase (NAMPT), the enzyme that catalyzes the rate-limiting step in the NAD^+^ salvage pathway, leads to toxic effects (Zabka et al., 2015). In summary, the pioneering work of other laboratories showed either complex phenotypes or the accumulation of toxic metabolites in mice with gene deletions of enzymes involved in the kynurenine pathway. Moreover, the existing genetic knockout models, unlike the ANDY mouse, are not “tunable,” and germline gene deletions can alter development, making phenotype analysis in adult mice even more complex. It is likely that the poor efficiency of tryptophan conversion typical of humans is also related to high ACMSD expression, potentially as a neuroprotective mechanism, which would make the ANDY mouse a rodent model potentially applicable to human metabolism. ANDY mice showed progressive and reversible blood NAD^+^ and NADP^+^ deficiency over time, which was highly significant as early as 3 weeks after transgene induction on the ND1 niacin-deficient diet. ANDY mice lost weight, with concurrent alterations in behavior, which may at least in part be explained by the NAD^+^ deficiency. NAD^+^ is an important coenzyme for the conversion of lactate to pyruvate, and the limited availability of NAD^+^ likely resulted in the observed altered lactate-to-pyruvate ratio. Because pyruvate is central to energy metabolism in the citrate cycle, reduced pyruvate levels may directly contribute to loss of body weight. In addition, disruption of NAD^+^ redox homeostasis and concurrent mitochondrial dysfunction have been associated with metabolic disorders, as recently reviewed by Cantó et al. (2015). NAD^+^ and acetyl-CoA are both co-substrates for enzymes that modulate the epigenome, such as sirtuins, PARPs, and histone acetyl transferases and other protein acetyl transferases. Changing either NAD^+^ levels or the relative availability of those two co-substrates may contribute to the altered epigenetic regulation of genes involved in the control of body weight and lipid metabolism, which is consistent with work by Cantó et al. (2009), Feige et al. (2008), Krishnan et al. (2012), and Verdin (2015). The observed shift in the NAD^+^/NADH ratio raises the possibility that niacin-deficient mice have a lowered oxidizing capacity, while the stable NADH levels suggest that reducing equivalents are still available for the electron transport apparatus with similar efficiency to the niacin-replete state.

In conclusion, the inducible ANDY mouse is a rodent model of dietary niacin dependence comparable to the human situation, with the potential to create varying NAD^+^ levels and NAD^+^ deficiency through dietary means. Phenotypic characterization of NAD^+^-deficient mice revealed weight loss, reduced liver pyruvate content indicative of low energy metabolism, and behavior changes, all of which are consistent with reduced whole-body niacin status. This model should be able to advance research on a vast array of pathologies associated with low NAD^+^ metabolism, NAD^+^-dependent DNA repair processes, carcinogenesis, epigenetic mark formation, behavioral changes linked to niacin deficiency, and other areas, including metabolic disorders and aging.

## STAR★METHODS

### CONTACT FOR REAGENTS AND RESOURCE SHARING

Further information and requests for resources and reagents should be directed to and will be fulfilled by the Lead Contact, Mirella L. Meyer-Ficca (Mirella.meyer@usu.edu).

### EXPERIMENTAL MODEL AND SUBJECT DETAILS

#### hACMSD Transgenic Mice

A tetracycline-responsive system that has a high amplitude of expression versus background transcription levels (Gossen et al., 1995; Meyer-Ficca et al., 2004) was selected for inducible expression of the human *ACMSD* (*hACMSD*) gene (Suppl. Figure S1). A cDNA clone (NCBI: BC107420, OpenBiosystems, GE Healthcare Dharmacon, Pittsburgh, PA, USA) encoding the human amino-carboxymuconate semialdehyde decarboxylase (*hACMSD*) coding sequence was amplified by PCR, sequence verified by conventional sequencing, and ligated into plasmid pBS31’RBGPA, a plasmid designed for FLPe recombination-based transgenesis (Beard et al., 2006). The resulting targeting vector contains the *hACMSD* cDNA under the control of a tetracycline-inducible CMV minimal promoter region. Co-transfection with plasmid pCAGGS-FlpE into the KH2 ES cell line was used to obtain the stable integration of a single copy of the transgenic expression cassette into safe-haven chromatin downstream of the *Col1a1* locus. The KH2 ES cell line also contains a single copy of the M2rtTA reverse tetracycline transactivator inserted into and under the transcriptional control of the ubiquitously expressed *ROSA26* locus and allows for dose-dependent tetracycline/DOX-inducible gene expression (Beard et al., 2006; Casola, 2010). Integration of the transgene was verified by Southern Blotting, PCR and inducible expression of the transgene in selected cell clones was verified using immunoblotting (Figure S1 b.). Transgenic animals were generated by ES cell injection into blastocysts. Offspring carrying the *hACMSD* transgene and the M2rtTA transactivator were identified by conventional genotyping using the following primers (hACMSD col/frt A1 (5′-GCA CAG CAT TGC GGA CAT GC-3′), hACMSD col/frt-B (5′-CCC TCC ATG TGT GAC CAA GG-3′), hACMSD col/frt C1 (5′-GCA GAA GCG CGG CCG TCT GG-3′), RoseCreERT2 A (5′-AAA GTC GCT CTG AGT TGT TAT-3′), RoseCreERT2 B (5′-GCG AAG AGT TTG TCC TCA ACC-3′), RoseCreERT2 C (5′-GGA GCG GGA GAA ATG GAT ATG-3′). The combination of primers A & B amplifies fragments indicating the presence of the transgene, the combination A & C amplifies a wild-type allele fragment. Transgenic offspring were backcrossed to C57BL/6J background (B6; The Jackson Laboratories, Bar Harbor, ME USA), and transgenic animals with the official designation C57BL/6J-Gt(Rosa)26Sor^tm1(rTTa*M2)Jae^Col1a1^tm6(tetO-hACMSD)MMF^ were maintained on a C57BL/6J background. All animals were maintained and experimental procedures were approved by Institutional Animal Care and Use Committees (IACUC) of Utah State University and of the University of Pennsylvania. Mice were bred and housed under standard housing conditions (group housing up to 5 mice per cage). For feeding trials, equal number of age and weight matched were randomly assigned for the experiments when they were between 7-14 weeks old. Unless indicated otherwise, male mice were used.

### METHOD DETAILS

#### hACMSD Transgene Induction and Feeding Regimens

Administration of DOX (DOX hyclate, Sigma Aldrich and Thermo Fisher Scientific) at 2 mg/ml in the drinking water induced transgene expression in mice. Animals were young adult mice ranging from 7-16 weeks of age with no differences in average age and body weight between the test groups at the beginning of each long-term feeding trials. Drinking water was changed every 48 hours. 48 hours after starting DOX application, animals were switched from chow to defined diets (see below, and Tables S1 and S2). Feed consumption was recorded three times weekly in 2-3 day intervals. Feed efficiency was calculated at gram of body weight gain per consumed calories and day.

#### Mouse feed composition

Standard chow diet was Teklad Rodent diet 8604 (24% crude protein, 63 mg/kg niacin, Envigo, Madison, WI, USA). Niacin-deficient diet (ND1) and control diet (CD1) were defined, purified diets that were specially compounded by Teklad laboratory animal diets (Envigo) as modifications of AIN-93G standard chow. Both, ND1 and CD1 contained 10% alcohol-washed casein as a vitamin-free protein source to limit tryptophan and niacin and were either supplemented with 30 mg/kg niacin (CD1) or without niacin supplementation (niacin-deficient, ND1). Detailed feed composition in Table S2. Feed intake was determined every 2-3 days by weighing feed added and leftover feed.

#### Development of Body Weight and Body Composition

Body weights were recorded three times per week (every 2-3 days). Body composition (body fat, lean mass, water) of each individual mouse in feeding trials was non-invasively analyzed using an MRI scanner (EchoMRI, Houston, USA) in biweekly intervals.

#### Open field test

Male mice were placed into an open field (40 cm^2^) for 5 minutes. A camera captured all horizontal movement and measured freezing. Movement through various pre-defined zones was analyzed automatically by the Any-maze software.

#### Locomotion test

Male mice were housed in Columbus Instruments’ Comprehensive Lab Animal Monitoring System with a home cage implementation (CLAMS-HC). After 3 hours of adjustment, ambulatory activity was measured as counts of break of neighboring infrared beams per measurement interval of 10 minutes for the duration of 24 hours. Any repeated interruptions of the same infrared beam was not scored as ambulatory counts. Activity data from CLAMS-HC were sub-grouped into light or dark periods and were analyzed with the CLAMS data examination Tool (CLAX;Columbus Instruments, Columbus OH). Results were shown for the measurements taken during the dark phase, which represents the active phase in nocturnal animals like mice.

#### Blood and Tissue Collection, Histology

For repeated-measurements at different time points, 50 μL aliquots of blood were obtained by facial vein puncture using 4 mm or 5 mm lancets (dependent on the age of the animal, Goldenrod, Medipoint), blood was heparinized, snap frozen on dry ice and stored at −80°C until further processing. At termination of the study, animals were euthanized, cardiac blood was collected and heparinized. Blood aliquots were snap frozen. Tissues were collected rapidly and snap frozen in liquid nitrogen and stored at −80°C. Tissues for histological analyses were fixed in Bouin’s solution, paraffin embedded and sectioned. Fresh tissue weights were determined for testes, perigonadal white adipose tissue and interscapular brown adipose tissue.

#### Immunoblot Analyses

Preparation of tissue lysates was performed as described earlier (Meyer-Ficca et al., 2011). The following antibodies were used: polyclonal rabbit anti-hACMSD (NBP1-33499, Novus Biologicals, Abnova), monoclonal mouse anti-α-Tubulin (TUBA, clone DM1A, T90626, Sigma-Aldrich). Secondary antibodies were donkey anti-mouse IgG, labeled with CY3 (Cat.-No. 715-165-150, Jackson ImmunoResearch, West Grove, PA, USA), donkey anti rabbit IgG, labeled with AF488 (Cat.-No. 711-546-152, Jackson ImmunoResearch). Fluorescence signals were captured using a Typhoon Fluorescence Scanner and ImageQuant TL software (both GE Healthcare).

#### NAD^+^ Quantification

Blood and tissue NAD^+^ was quantified using a method described by (Jacobson and Jacobson, 1997; Shah et al., 1995), using chemicals from Sigma, Aldrich (St Louis, MO, USA), and assayed on a SpectraMax Plus 384 plate reader (Molecular Devices, Sunnyvale, CA, USA). Briefly, 55 μl of heparinized whole blood or 30-40 mg of frozen tissue powder were lysed in exactly 10 volumes of 1 M NaOH. The pH was adjusted to pH 7.0 immediately with 2 M H_3_PO_4_, followed by addition of 11 volumes of 1 M HClO_4_ and a 10 min incubation step on ice. After a subsequent centrifugation (2000 x g, 5 minutes), 2 volumes of supernatant were mixed with 1 volumes of 1 M KOH, incubated on ice for 10 minutes, centrifuged (850 x g, 10 minutes), and the supernatant was used for immediate NAD^+^/NADP^+^ quantification. Alternatively, aliquots of the supernatant were stored at −80°C until use. For the NAD^+^/NADP^+^ Microplate assay, quadruplets of each sample (50 μl lysate, diluted with 50 μl H_2_O) were measured in a 96-well plate format. 50 μl of NAD^+^ mix (10 M EtOH, 1.2 M bicine, 10 mM thiazolyl Blue, 0.2 M EDTA, 100 mg/ml bovine serum albumine, 40 mM phenazine ethosulfate and 1 mg/ml alcohol dehydrogenase in 0.1 M bicine) or NADP^+^ mix (0.2 M isocitrate, 1 M phosphate buffer pH 6.8, 10 mM thiazolyl blue, 0.2 M MgCl2, 100 mg/ml bovine serum albumine, 40 mM phenazine ethosulfate, 10 mg/ml isocitrate dehydrogenase) were added to each well to quantify NAD^+^ or NADP^+^, respectively. The absorbance at 570 nm was measured immediately after addition of NAD mix and every 10 minutes for about 1 hour. The increase in absorbance over time was used to calculate concentrations. The niacin number of animals was calculated as a measure of niacin status using the following formula: (NAD^+^ / [NAD^+^ + NADP^+^])x 100% (Jacobson, 1993; Jacobson and Jacobson, 1997), and then converted to relative niacin indices by dividing the niacin number of each sample by the average niacin numbers obtained in control animals on niacin replete diet (CD1).

#### Metabolomics Analysis

Metabolomics analysis was performed at the Metabolomics Core Facility at the University of Utah.

##### 1. Metabolite Extraction

Liver samples were kept at −80°C prior to analysis. Frozen liver samples were transferred to labeled bead tubes and weighed. To each tube, 450 μL of ice-cold 90% methanol solution containing the internal standards (1 μg of d4-succinic acid and 5 μg of labeled amino acids (13C, 15N–labeled) mixture) were added. Using an OMNI Bead Ruptor, samples were homogenized at 6.45 MHz for 30 s and then incubated at −20°C for 1 hour. After incubation, samples were centrifuged at 20,000 x g for 5 minutes at 4°C and the supernatant transferred to fresh tubes. Quality controls were generated by collecting and combining 15% volume of each sample into a single tube. Samples were dried in a Speed-Vac vacuum centrifuge.

##### 2. Gas Chromatography-mass spectrometry (GC-MS) analyses:

GC-MS analyses were performed with a Waters GCT Premier mass spectrometer fitted with an Agilent 6890 gas chromatograph and a Gerstel MPS2 auto sampler. Dried samples were suspended in 40 μL of 40 mg/mL O-methoxylamine hydrochloride (MOX) in pyridine and incubated for one hour at 30°C. To each sample of 25 μL, 40 μL of N-methyl-N-trimethylsilyltrifluoracetamide (MSTFA) was added automatically and incubated for 1h at 37°C with shaking. After incubation, 3 μL of a fatty acid methyl ester standard (FAMES) solution was added, then 1 μL of the prepared sample was injected to the gas chromatograph inlet in the split mode with the inlet temperature held at 250°C. A 10:1 split ratio was used for analysis. The gas chromatograph had an initial temperature of 95°C for one minute, followed by a 40°C/min ramp to 110°C and a hold time of 2 minutes. This was followed by a second 5°C/min ramp to 250°C, a third ramp to 350°C, then a final hold time of 3 minutes. A 30 m Phenomex ZB5-5 MSi column with a 5 m long guard column was employed for chromatographic separation. Helium was used as the carrier gas at 1 mL/min. Due to the high amounts of several metabolites, all samples were also analyzed once more at a 10-fold dilution. Data was collected using MassLynx 4.1 software (Waters). Metabolites were identified and their peak area was recorded using QuanLynx. Metabolite identity was established using a combination of an in house metabolite library developed using pure purchased standards and the commercially available NIST library. Data were transferred to an Excel spread sheet (Microsoft, Redmond WA), and subsequent analysis was performed using Metaboanalysist 3.0 software (www.metaboanalyst.ca).

#### Acetyl Coenzyme A Quantification

Acetyl Coenzyme A was quantified using an Acetyl Coenzyme A Assay kit (Sigma Aldrich) according to the manufacturer’s instructions.

#### Histological Sections

Histological sections as well as hematoxylin and eosin staining was performed by the Utah Veterinary Diagnostic Laboratory using standard methods. Grayscale images were taken on a AX10 scope.A1 microscope (Zeiss) equipped with a computer-controlled charge coupled device (CCD) black-and-white camera (Zeiss Axiocam).

### QUANTIFICATION AND STATISTICAL ANALYSIS

#### Statistical Analysis

Data are presented as means ± SD. Comparisons between two groups were performed by Student’s t tests, and multiple t tests with Benjamini two-stage linear step up. Comparison between 3 and more groups by one-way ANOVA with Tukey’s multiple comparison tests or with post-test for linear trend. P values are as follows: * indicates p < 0.05, ** p < 0.01, *** p < 0.001, **** p < 0.0001. Statistical parameters used are indicated in each figure legend. Statistical evaluation was performed with Prism GraphPad 6.0 and 7.0 (Graph-Pad Software, LA Jolla, CA, USA).

## Supplementary Material

1

## Figures and Tables

**Table:** KEY RESOURCES TABLE

REAGENT or RESOURCE	SOURCE	IDENTIFIER
Antibodies		
Rabbit anti ACMSD (specific for human and mouse)	Novus Biologicals	Cat# NBP1-33499; RRID:AB_2223750
Mouse anti ACMSD (specific for human)	Abnova	Cat# H00130013-A01; RRID: AB_463516
Mouse anti alpha-Tubulin	Sigma Aldrich	Clone DM1A, Cat#T6199; RRID:AB_477583
Donkey anti-mouse IgG, labeled with CY3	Jackson ImmunoResearch	Cat# 715-165-150; RRID:AB_2340813
Donkey anti rabbit IgG, labeled with AF488	Jackson ImmunoResearch	Cat# 711-546-152; RRID:AB_2340619
Chemicals, Peptides, and Recombinant Proteins		
Bouin’s solution	Sigma Aldrich	HT10132
Doxycyline Hyclate	Sigma Aldrich	D9891
Doxycyline Hyclate	Alfa Aesar	J6057922
NaOH	Sigma Aldrich	S5881
H_3_PO_4_	BioUltra	79617
HClO_4_	Sigma Aldrich	77234
KOH	EMD Millipore	1.05012
Ethanol	Sigma Aldrich	E7023
Bicine	Sigma Aldrich	B3876
Thiazolyl Blue	Sigma Aldrich	M2128
EDTA Disodium	EMD Millipore	324503
Bovine Serum Albumin	Sigma Aldrich	A7906
Phenazine Ethosulfate	Sigma Aldrich	P4544
Alcohol Dehydrogenase	Sigma Aldrich	A3263
Isocitrate	Sigma Aldrich	I1252
Isocitrate Dehydrogenase	Sigma Aldrich	I1877
NAD (β-NAD^+^)	Sigma Aldrich	N0632
NADP (β-NADP^+^)	Sigma Aldrich	N8035
Critical Commercial Assays		
Acetyl Coenzyme A Assay	Sigma Aldrich	MAK039
Experimental Models: Cell Lines		
KH2 mouse embryonic stem cell line		(RRID:CVCL_C317)
Experimental Models: Organisms/Strains		
*Mus musculus* / C57BL/6J-Gt(Rosa)26Sor^tm1(rTTa*M2)Jae^Col1a1^tm6(tetO-hACMSD)MMF^	This Study	NA
C57BL/6J	Jackson Laboratories	Stock No: 000664
Oligonucleotides		
ACMSD col/frt A1 (5′-GCA CAG CAT TGC GGA CAT GC-3′)	(Beard et al., 2006)	NA
hACMSD col/frt-B (5′-CCC TCC ATG TGT GAC CAA GG-3′)	(Beard et al., 2006)	NA
hACMSD col/frt C1 (5′-GCA GAA GCG CGG CCG TCT GG-3′)	(Beard et al., 2006)	NA
RoseCreERT2 A (5′-AAA GTC GCT CTG AGT TGT TAT-3′)	(Beard et al., 2006)	NA
RoseCreERT2 B (5′-GCG AAG AGT TTG TCC TCA ACC-3′)	(Beard et al., 2006)	NA
RoseCreERT2 C (5′-GGA GCG GGA GAA ATG GAT ATG-3′)	(Beard et al., 2006)	NA
Recombinant DNA		
Aminocarboxymuconate semialdehyde decarboxylase (hACMSD) coding sequence	Open Biosystems	NCBI: BC107420, NCBI: 130013, Clone ID: 4594027
pBS31’RBGPA	(Beard et al., 2006)	NA
pCAGGS-FlpE	(Beard et al., 2006)	NA
Software and Algorithms		
ImageQuant TL	GE Healthcare	NA
MassLynx4.1 software	Waters	NA
QuanLynx	Waters	NA
Metaboanalysist 3.0		www.metaboanalyst.ca
Oxymax 5.43	Columbus Instruments	http://www.colinst.com/
CLAX	Columbus Instruments	http://www.colinst.com/
ANY-maze behavior tracking software	Stoelting	https://www.stoeltingco.com/anymaze.html
Prism 6.0 and 7.0	GraphPad	https://www.graphpad.com/scientific-software/prism/
Other		
Niacin deficient ND1	Envigo	TD.140376
Niacin Replete CD1	Envigo	TD.140375
Niacin Deficient ND2	Envigo	TD.150137
Niacin replete CD2	Envigo	TD.150136

## Highlights

- A rodent model with niacin dependence and tryptophan conversion similar to humans
- Niacin-deficient diets can induce NAD^+^ deficiency
- NAD^+^ deficiency depends on tryptophan content in diet
- NAD^+^-deficient mice have adverse health effects
