# Supplementary material for: Alpha-Amino-Beta-Carboxy-Muconate-Semialdehyde Decarboxylase Controls Dietary Niacin Requirements for NAD+ Synthesis
Source: Cell Rep. Author manuscript; Available in PMC 2023 Jan 1. (PMC9805792; doi:10.1016/j.celrep.2018.09.091)
Supplement: 1 [file NIHMS1856803-supplement-1.pdf]

Cell Reports, Volume *25*

Supplemental Information

Alpha-Amino-Beta-Carboxy-Muconate-Semialdehyde Decarboxylase Controls Dietary Niacin Requirements for NAD^+ Synthesis

Laura Palzer, Jessica J. Bader, Frances Angel, Megan Witzel, Sydney Blaser, Alexis McNeil, Miles K. Wandersee, N. Adrian Leu, Christopher J. Lengner, Clara E. Cho, Kevin D. Welch, James B. Kirkland, Ralph G. Meyer, and Mirella L. Meyer-Ficca

A

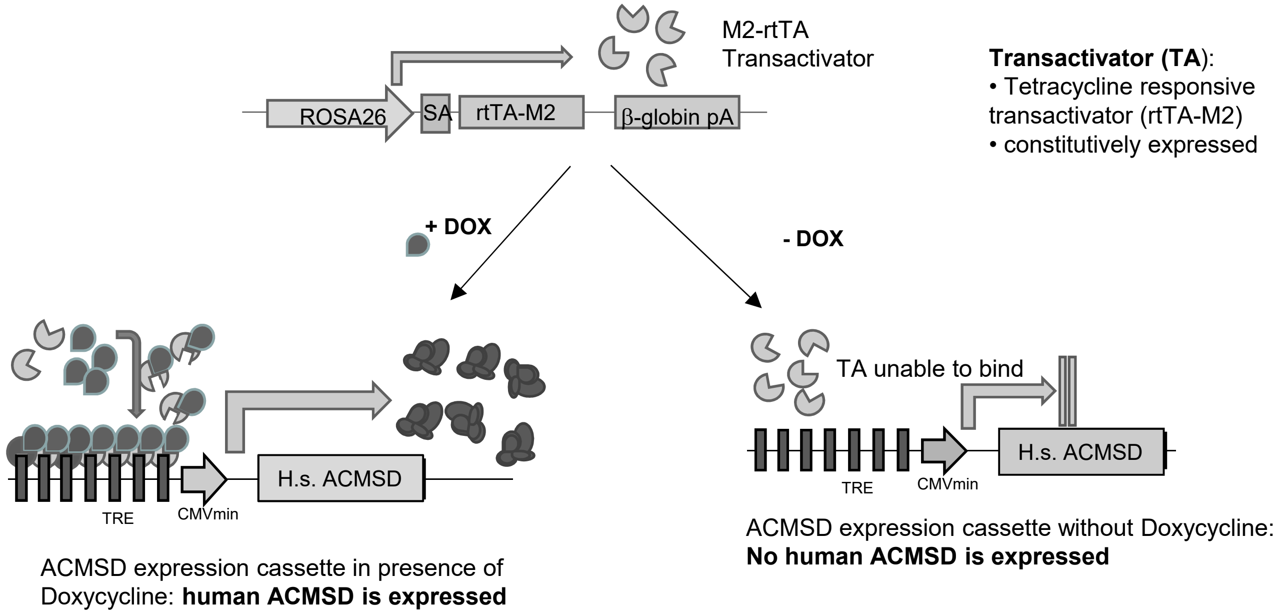

B

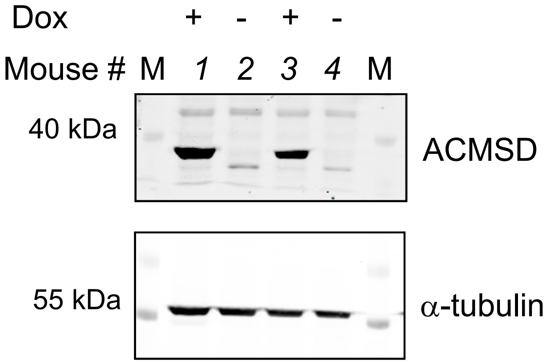

Figure S1, related to Figure 2. (A) Mode of operation of the ACMSD overexpression system. The M2rtTA transactivator is constitutive expressed in all transgenic tissues. In the presence of doxycycline, the transactivator binds to the tetracycline responsive elements (TRE) and activates expression of ACMSD (left). Without doxycycline, the transactivator is unable to elicit transcription of the ACMSD transgene (right).
(B) **ACMSD expression.** Western Blot of liver tissue obtained from transgenic animals after administration of 2 mg/ml doxycycline in their drinking water for 48 hours. ACMSD signals of ~38 kDa indicate transgene expression induced by doxycycline administration, while no ACMSD was detectable in tissues from animals not induced with doxycycline. Tissues were taken from animals that had been on either doxycycline containing water Niacin content in the diet

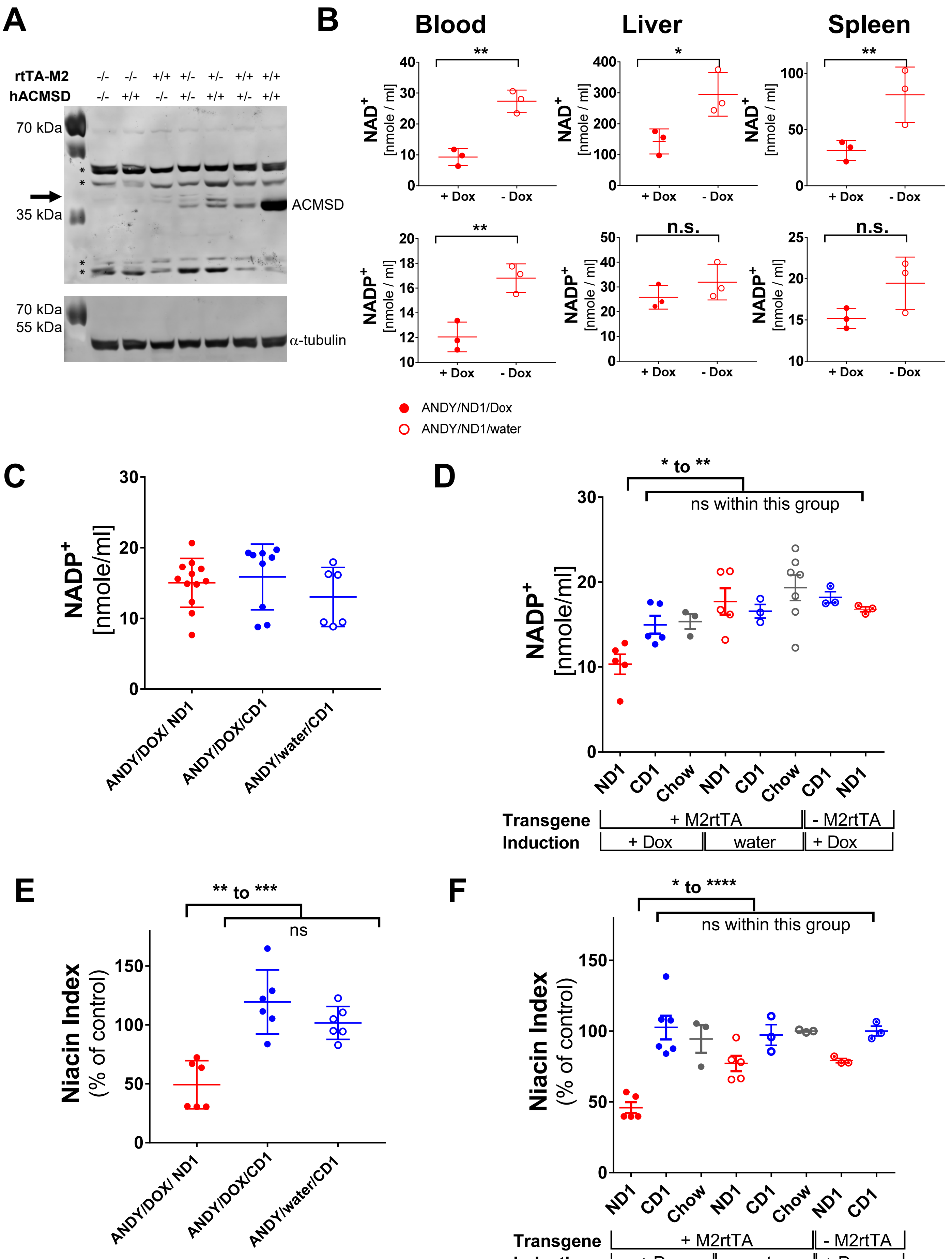

Figure S2, related to Figure 2

Figure S2, related to Figure 2:
(A) Expression level of ACMSD increases with copy number of transgenes and M2rtTA transactivator. ACMSD expression in liver tissue from animals carrying the indicated transgene composition after doxycycline application was analyzed by Western blot with a rabbit anti ACMSD antibody. No ACMSD transgene expression is seen in absence of the transactivator transgene (lane 2 and 3) independent of ACMSD transgene presence and induction, indicating that there is no leakiness of the system. Increased copy numbers of either transactivator or ACMSD transgene correlate with increased amounts of detectable ACMSD, and highest ACMSD was detectable when two transgenes of each were present (right lane); arrow = ACMSD specific signals at ~38 kDa , * = unspecific bands;. ACMSD signals indicate transgene expression, TUBA signals indicate equal loading.
(B) Animals fed niacin free ND1 diet had significantly lower NAD^+ tissue content when the ACMSD transgene was expressed (+ Dox) compared to animals without ACMSD transgene expression (- Dox). $NADP^+$ content was significantly reduced in blood, but not in liver or spleen. Analyzed tissues are indicated above. Significance was tested using Student's t test; n= 3 male mice.
(C) Blood $NADP^+$ levels after three weeks on the indicated diets. n = 6 to 12 animals per diet group, from three independent experiments. (D) Blood $NADP^+$ content after 3-4 months on diets. Significance was tested by 1-way ANOVA and Tukey's multiple comparison test; n= 3-20 male mice per group from 4 independent experiments.
(E) and (F) Niacin indices in animals on the indicated feed group for 3 weeks (E) or 4 months (F). Blood NAD^+ and $NADP^+$ levels (shown in figures 2C and 2D and figures S2C and S2D) were used to calculate individual "niacin indices" (100* [NAD^+]/([NAD^+ +$NADP^+$]). Normalization of niacin numbers to the average niacin number observed in ANDY/water/CD1 resulted in the niacin index. Significance was tested by 1-way ANOVA and Tukey's multiple comparison test; n= 3-20 male mice per group from 4 independent experiments

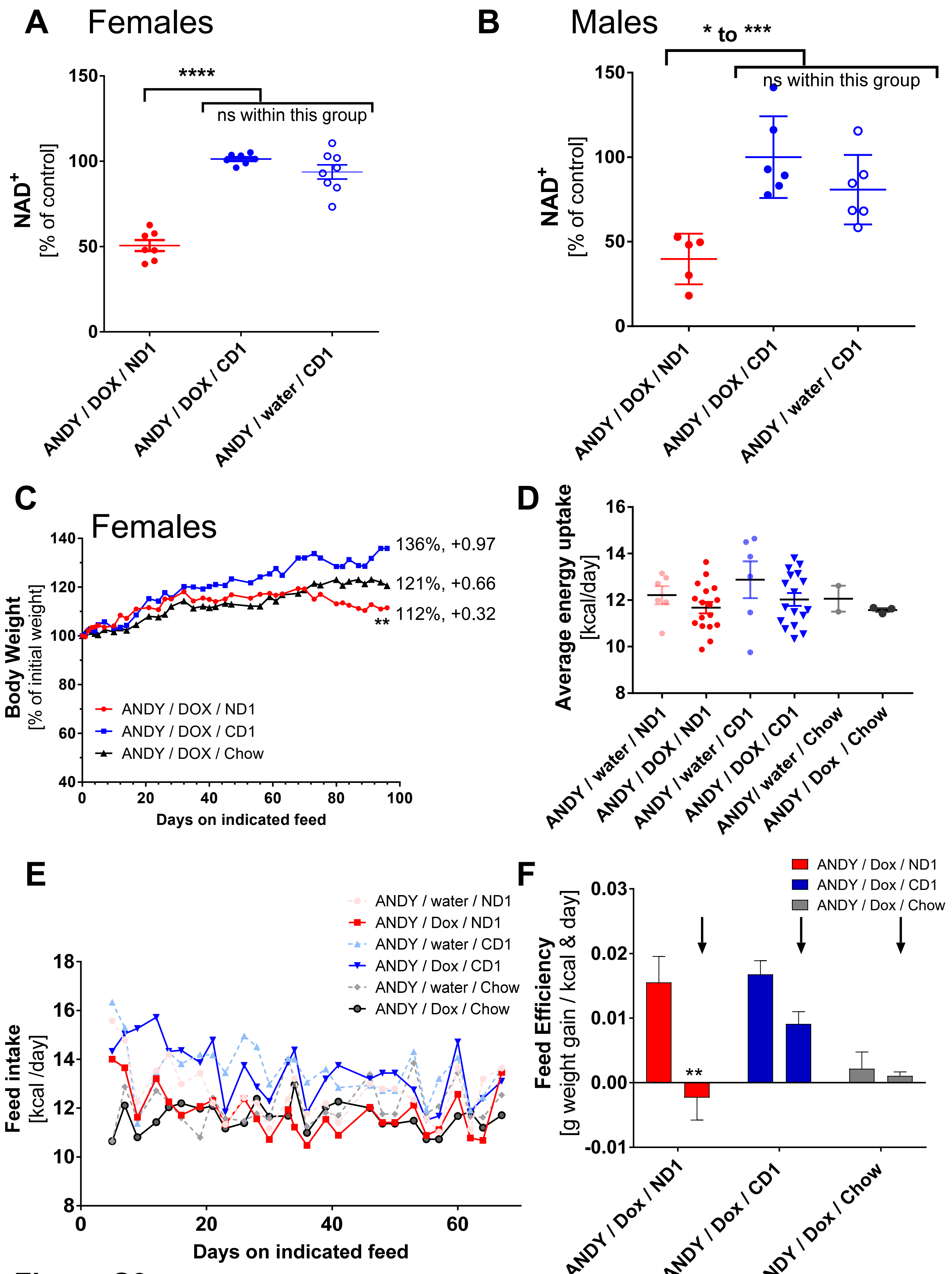
A
Females
B
Males
****
ns within this group
* to ***
NAD+ [% of control]
150
100
50
0
ANDY / DOX / ND1
ANDY / DOX / CD1
ANDY / water / CD1
C
Females
Body Weight [% of initial weight]
136%, +0.97
121%, +0.66
112%, +0.32
**
ANDY / DOX / ND1
ANDY / DOX / CD1
ANDY / DOX / Chow
Days on indicated feed
D
Average energy uptake [kcal/day]
ANDY / water / ND1
ANDY / DOX / ND1
ANDY / water / CD1
ANDY / DOX / CD1
ANDY/ water / Chow
ANDY / Dox / Chow
E
ANDY / water / ND1
ANDY / Dox / ND1
ANDY / water / CD1
ANDY / Dox / CD1
ANDY / water / Chow
ANDY / Dox / Chow
Feed intake [kcal /day]
Days on indicated feed
F
ANDY / Dox / ND1
ANDY / Dox / CD1
ANDY / Dox / Chow
Feed Efficiency [g weight gain / kcal & day]
**

Figure S3

Figure S3, Related to Figure 2 and to Figure 4. Despite comparable drop in blood NAD levels, effects of niacin deficient diet on body weight and composition was less pronounced in female mice (A) Blood NAD^+ content in female ANDY / DOX/ ND1 (mice with transgene induction kept on niacin deficient CD1 diet for 9-14 weeks) was significantly lower than of blood NAD^+ in ANDY/DOX/CD1 (controls kept on a niacin replete diet (CD1)) or controls without transgene induction (ND1 water). Data were obtained in two independent studies and is shown as % of average ANDY/DOX/CD1 value, n=7-8 animals. Significant was tested using 1-way ANOVA with Tukey multiple comparison, **** indicates $p < 0.0001$.

(B) Blood NAD^+ content in male mice with transgene induction kept on niacin deficient diet for 3 weeks was significantly lower than blood NAD^+ in controls kept on a niacin replete diet (CD1) or controls without transgene induction (CD1 water). Data were obtained in two independent studies and is shown as % of average ANDY/DOX/CD1 value, n= 5-6 animals. Significant was tested using 1-way ANOVA with Tukey multiple comparison, , **, $p < 0.01$, ***, $p < 0.001$.

(C) Female body weight development over a time frame of 96 days on indicated diet and doxycycline. In the presence of transgene induction, female mice on the niacin deficient diet ND1 displayed a lower trend for body weight gain compared to animals fed either niacin replete control diet CD1 or chow, with a slope of +0.32 for niacin deficient diet, +0.66 for chow and +0.97 for CD1. (Repeated measures 1-Way ANOVA with post test for linear trend for each feed group (n= 5 animals per group). Asterisk indicates the first point of significant difference between induced ND1 and control diet CD1 (in individual t tests).

(D) Average energy intake of male animals on the indicated diets. There was no significant difference in calorie intake between animals on the indicated diets, despite a tendency for the animals on ND1 and Dox to eat less. Data were combined from three independent feeding trials. Individual feed consumption was determined three times a week over a period of 6-10 weeks and the average daily calorie consumption was calculated. 1-Way ANOVA analysis, n= 2-16 animals per group.)

(E) Average daily calorie consumption over the course of a 67 day feeding trial of male ANDY mice. Feed intake was measured in 2-3 day intervals and daily calorie intake was calculated. No significant feed-dependent variation was found (2-Way ANOVA with Tukey's multiple comparison test, n=3 animals per group)

(F) Feed efficiency in male mice measured as daily weight gain per consumed calories. While feed efficiency at the beginning of the feeding trial was comparable in all feed groups, a significant decrease in feed efficiency was observed in male ANDY mice that had been on ND1 for several weeks. The left columns show average daily body weight gain per consumed calories observed during the initial three weeks of a feeding trial, the right columns (marked with arrows) show body weight gain during a later phase of the feeding trial (weeks 6-8). 2-way ANOVA, n=3-6 animals, Sidak's Multiple Comparison Test, , **, $p < 0.01$.

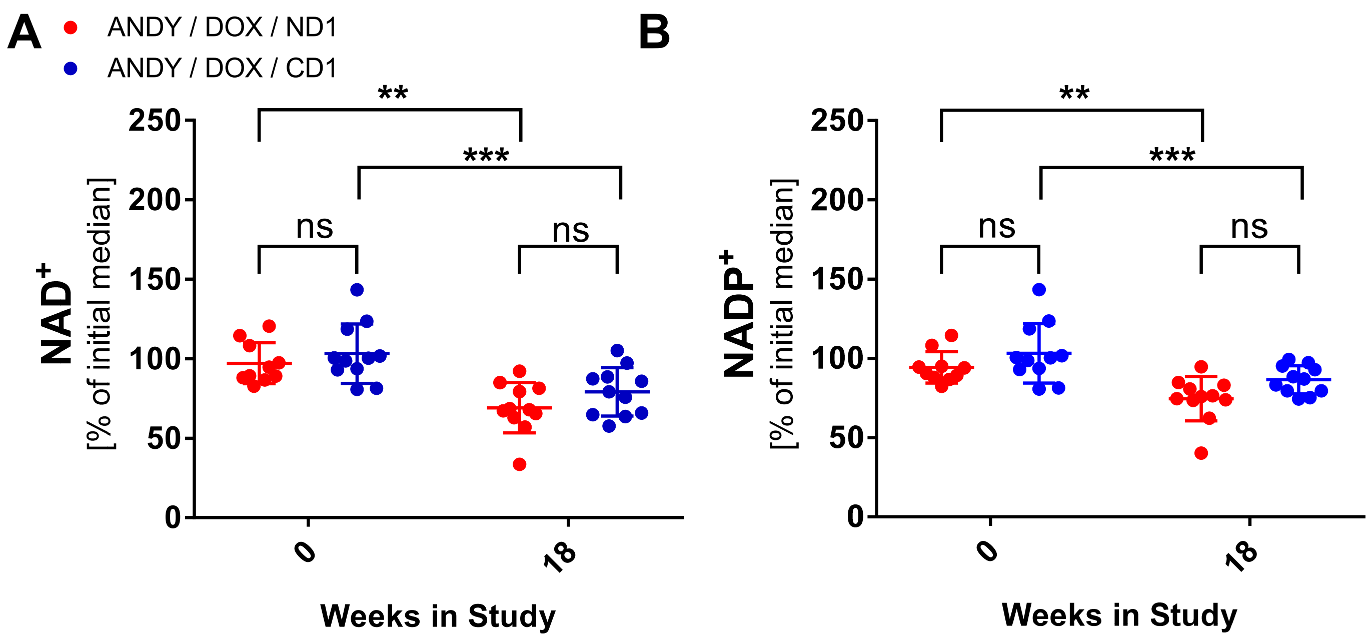

Figure S4, related to Figure 3:

After feeding for 16 weeks on niacin-deficient (ND1) or -replete (CD1) diets, all animals received niacin containing control diet for 2 weeks prior blood collection to reverse niacin deficiency. At this point, ND1 and CD1 animals no longer showed significantly different total NAD^+ (A) or $NADP^+$ (B) levels. However, all animals had lower average blood NAD^+ and $NADP^+$ values than at the outset of the trials 18 weeks prior. Significance was analyzed using paired (within feed groups) and unpaired (between feed groups) Student's t-test; n = 11 males.

Table S1, related to Figure 2: Macronutrient composition of diets.

	CD1	ND1	Chow
**Niacin [mg/kg]**	30	0	63
**Calorie density [kcal/g]**	3.8	3.8	3.0
**% kCal from …**			
**…Protein**	9.8	9.8	32
**…Carbohydrate**	73.4	73.4	54
**…Fat**	16.8	16.8	14

Table S2, related to Figure 2: Detailed composition of the diets; all are AIN-93G based. [g/kg]

Component	Concentration [g/kg]			
	**CD1**	**ND1**	**CD2**	**ND2**
Casein, “vitamin-Free”	100	100	200	200
L-Cystein	2.3	2.3	2.3	2.3
Corn Starch	514	514	414	414
Maltrodextrin	132	132	132	132
Sucrose	100	100	100	100
Soybean Oil	70	70	70	70
Cellulose	50	50	50	50
Mineral Mix, w/o Ca & P	13.39	13.39	13.39	13.39
Calcium Carbonate,	5.1	5.1	7.4	7.4
Calcium Phosphate, dibasic	10.0	10.0	6.8	6.8
**Niacin**	**0.03**	**0**	**0.06**	**0**
Calcium Pantothenate	0.016	0.016	0.016	0.016
Pyridoxine HCl	0.007	0.007	0.007	0.007
Thiamin HCl	0.006	0.006	0.006	0.006
Riboflavin	0.006	0.006	0.006	0.006
Folic Acid	0.002	0.002	0.002	0.002
Biotin	0.0002	0.0002	0.0002	0.0002
Vitamin B12 (0.1% in mannitol)	0.025	0.025	0.025	0.025
Vitamin E (DL-alpha tocopheryl acetate, 500 IU/g)	0.15	0.15	0.15	0.15
Vitamin A Palmitate (500,000 IU/g)	0.008	0.008	0.008	0.008
Vitamin D3 (Cholecalciferol, 500,000 IU/g)	0.002	0.002	0.002	0.002
Vitamin K1 (Phylloquinone)	0.001	0.001	0.001	0.001
Choline Bitartate	2.5	2.5	2.5	2.5
TBHQ, antioxidant	0.014	0.014	0.014	0.014

Table S3, related to Figure 3. Development of blood NAD values and significance over time. (Multiple t tests with Two-stage step-up procedure of Benjamini, Krieger and Yekutiely, with Q =1%)

**NAD**	**ANDY/DOX/ND1**			**ANDY/DOX/CD1**			**Multiple t-test**[1)]	**P value**
**Weeks on diet**	Mean	SD	N	Mean	SD	N	No	
**0**	97.1258	12.9313	10	103.198	18.6913	11	No	P = 0.221
**6**	53.4375	12.1363	11	98.1452	8.21351	11	Yes (***)	$P < 2.59 * 10^{-9}$
**16**	38.6188	7.21962	11	93.7224	12.6512	11	Yes (***)	$P < 6.16 * 10^{-11}$
**18**	69.1307	15.7941	11	79.1573	15.1509	11	No	0.282

Table S4, related to Figure 3. Development of blood NADP values and significance over time. (Multiple t tests with Two-stage step-up procedure of Benjamini, Krieger and Yekutiely, with Q =1%)

	**ANDY/DOX/ND1**			**ANDY/DOX/CD1**			**Multiple t-test**	**P value**
**Weeks on diet**	Mean	SD	N	Mean	SD	N	No	
**0**	94.3031	9.91764	10	103.198	18.6913	11	No	P = 0.489
**6**	84.1820	8.72605	11	100.004	7.03731	11	Yes (***)	$P < 1.4 * 10^{-4}$
**16**	72.9344	8.55892	11	102.921	6.18146	11	Yes (***)	$P < 8.6 * 10^{-9}$
**18**	74.5296	13.9903	11	86.5088	8.86119	11	No	P = 0.0263
